# Genome Wide DNA Copy Number Analysis of Serous Type Ovarian Carcinomas Identifies Genetic Markers Predictive of Clinical Outcome

**DOI:** 10.1371/journal.pone.0030996

**Published:** 2012-02-15

**Authors:** David A. Engler, Sumeet Gupta, Whitfield B. Growdon, Ronny I. Drapkin, Mai Nitta, Petra A. Sergent, Serena F. Allred, Jenny Gross, Michael T. Deavers, Wen-Lin Kuo, Beth Y. Karlan, Bo R. Rueda, Sandra Orsulic, David M. Gershenson, Michael J. Birrer, Joe W. Gray, Gayatry Mohapatra

**Affiliations:** 1 Department of Pathology, Massachusetts General Hospital, Boston, Massachusetts, United States of America; 2 Department of Statistics, Brigham Young University, Provo, Utah, United States of America; 3 Whitehead Institute of Biomedical Research, Cambridge, Massachusetts, United States of America; 4 Department of Vincent Obstetrics and Gynecology, Vincent Center for Reproductive Biology, Massachusetts General Hospital, Boston, Massachusetts, United States of America; 5 Department of Medical Oncology, Dana-Farber Cancer Institute, Boston, Massachusetts, United States of America; 6 Life Sciences Division, Lawrence Berkeley National Laboratory, Berkeley, California, United States of America; 7 Women's Cancer Research Institute, Cedars-Sinai Medical Center, Los Angeles, California, United States of America; 8 Department of Pathology and Gynecology Oncology, The University of Texas MD Anderson Cancer Center, Houston, Texas, United States of America; 9 Center for Cancer Research, Massachusetts General Hospital, Boston, Massachusetts, United States of America; Ohio State University Medical Center, United States of America

## Abstract

Ovarian cancer is the fifth leading cause of cancer death in women. Ovarian cancers display a high degree of complex genetic alterations involving many oncogenes and tumor suppressor genes. Analysis of the association between genetic alterations and clinical endpoints such as survival will lead to improved patient management via genetic stratification of patients into clinically relevant subgroups. In this study, we aim to define subgroups of high-grade serous ovarian carcinomas that differ with respect to prognosis and overall survival. Genome-wide DNA copy number alterations (CNAs) were measured in 72 clinically annotated, high-grade serous tumors using high-resolution oligonucleotide arrays. Two clinically annotated, independent cohorts were used for validation. Unsupervised hierarchical clustering of copy number data derived from the 72 patient cohort resulted in two clusters with significant difference in progression free survival (PFS) and a marginal difference in overall survival (OS). GISTIC analysis of the two clusters identified altered regions unique to each cluster. Supervised clustering of two independent large cohorts of high-grade serous tumors using the classification scheme derived from the two initial clusters validated our results and identified 8 genomic regions that are distinctly different among the subgroups. These 8 regions map to 8p21.3, 8p23.2, 12p12.1, 17p11.2, 17p12, 19q12, 20q11.21 and 20q13.12; and harbor potential oncogenes and tumor suppressor genes that are likely to be involved in the pathogenesis of ovarian carcinoma. We have identified a set of genetic alterations that could be used for stratification of high-grade serous tumors into clinically relevant treatment subgroups.

## Introduction

Epithelial ovarian carcinoma represents the fifth leading cause of cancer death among women in the United States [1], [2]. It is estimated that there will be 21,550 cases of invasive ovarian cancer diagnosed and 14,660 deaths attributed to ovarian cancer in 2009 [3]. The five year survival rate of ovarian cancer ranges from 30 to 92%, depending on the spread of the disease at the time of diagnosis [3]. While early-stage ovarian cancers are highly curable, over 70% of ovarian cancer patients are diagnosed with the advanced disease with lower cure rates and are associated with significant morbidity and mortality [4]. Over the past decades there have been significant advances in ovarian cancer treatment as a result of improved surgical techniques and chemotherapy regimens through multiple clinical trials [5], [6]. Debulking surgery has become the standard treatment for advanced stage ovarian carcinoma; a residual tumor size of greater than 2 cm is associated with a survival of 12–16 months, compared with 40–45 months if the tumor is less than 2 cm [7], [8]. Adjuvant chemotherapy with platinum and taxane based regimens improves both disease free survival and overall survival in all patient subgroups; however, the longest survival periods are observed in optimally debulked patients. Up to 80% of patients with advanced stage disease experience an initial response to chemotherapy but eventually relapse with a median progression free survival of 18 months [9], [10], [11], [12], [13]. A number of resistance mechanisms have been defined in vitro [14], [15], [16]. However, the importance of these resistance mechanisms in patients remains unclear. Thus, there is a need for improvement in the understanding of the underlying genetic alterations involved in the pathogenesis of ovarian cancer. Identification of prognostic/predictive markers can improve patient management and allow development of molecularly targeted therapeutics.

The serous type ovarian carcinoma accounts for approximately 70% of ovarian cancer cases and is one of the clinically aggressive subtypes [17]. High-grade serous tumors differ from all other ovarian carcinomas in terms of their pathology, pathogenesis, prognosis and underlying genetic alterations [18], [19]. The most frequently documented mutation is in the *TP53* tumor suppressor gene.

Expression profiling-based studies have also shown that high-grade tumors cluster separately from low grade carcinomas and borderline tumors [20], [21]. Several expression profiling based studies have identified gene expression signatures associated with response to chemotherapy [22], [23] and to different subtypes of ovarian cancer [21], [24]. High-level amplifications of *ERBB2*, *MYC*, *PIK3CA*, *EVI1*, *RAB25*, *AKT2*, *CCNE1*, *NOTCH3*, *FGFR2*, *CCND1*, *PAK1*, *EMSY*, *ZNF217*, *NCOA3* [23], [25], [26], [27], [28], [29], [30], [31], [32] and homozygous deletion, mutation, reduced expression and/or hypermethylation of *TP53*, *KRAS*, *LOT1*, *DOC2*, *NOEY2*, *OVCA1*, *SPARC*, *CDKN2A*, *RB1*, *PTEN* [33], [34], [35], [36], [37], [38], [39] genes have also been reported. However, little consensus or overlap between all these studies has emerged.

Array-based comparative genomic hybridization (aCGH) allows detection of DNA copy number alterations (CNA) and provides a global assessment of molecular events in the genome [40]. Several studies have been reported utilizing either conventional metaphase chromosome-based CGH [41], [42], [43] or array-based high resolution genomic technologies for identifying genome wide CNAs in ovarian cancer [23], [44], [45], [46], [47]. The above mentioned studies have identified frequent regions of increased copy number along 1q, 3q26, 7q32–q36, 8q24, 17q32 and 20q13; and regions of decreased copy number along 1p36, 4q, 13q, 16q, 18q and Xq12. However, specific genetic markers that are predictive of clinical outcome are yet to be identified for high-grade ovarian cancers. The rationale for our study is based on the idea that genetic alterations are the cause of tumor development and progression. Therefore, it is likely that combination of specific genetic alterations will be predictive of clinical behavior [48], [49]. In this study, using high-resolution aCGH, we sought to identify potentially useful DNA-based prognostic marker/s to delineate high-grade serous type ovarian cancer patients into molecularly defined clinically relevant subgroups.

## Materials and Methods

### Tumor samples and clinical data

The study group included tumor samples from 72 patients identified within prospectively collected MGH Gynecological Tissue Repository and Cedars-Sinai Women's Cancer Research Program Tissue Bank under IRB approved protocols at Massachusetts General Hospital and Cedars-Sinai Medical Center from 1991 to 2008 (Table 1). Under these protocols, patients with suspected ovarian cancer are consented in writing for tissue collection and prospective clinical data collection prior to surgical exploration. Frozen tumor tissues were collected, catalogued and anonymized. In each case, a small piece of tissue adjacent to the tissue that was used for DNA extraction, was paraffin embedded and H&E stained for histological validation. All samples were reviewed by a pathologist to confirm the presence of viable tumor cells in the tissue sample. Only samples with more than 70–80% viable tumor tissue were chosen for this study. Clinical data were then paired to the assigned catalogue number of each sample. Clinical factors including age at diagnosis, stage of disease, grade of tumor, origin of tumor (ovary, peritoneum, fallopian tube), specific surgical therapy, specific chemotherapy, platinum sensitivity, recurrence, progression free survival (PFS), overall survival (OS) were recorded and paired with the molecular data for correlation.

**Table 1:** Patient characterisitics.

Age		
	Median	60.1
	Range	36.9, 90.5
Grade		
	2	5 (6.9%)
	3	67 (93.0%)
Stage		
	II	5 (6.9%)
	III	43 (59.7%)
	IV	24 (33.3%)
Primary tumor site		
	ovary	56 (77.7%)
	peritoneal	12 (16.7%)
	fallopian tube	4 (5.6%)
Radiation		
	Yes	5 (6.9%)
	No	67 (93.0%)
Optimal Cytoreduction		
	Yes	64 (88.9%)
	No	8 (11.1%)
Platinum Sensitive		
	Yes	39 (54.2%)
	No	27 (37.5%)
	Not available	6 (8.3%)
Bowel Resection		
	Yes	21 (29.2%)
	No	51(70.8%)
Overall Survival		
	median (months)	38.5
	% censoring	59.2
	hospice/deceased	29 (40.3%)
Progression-free Survival		
	median (months)	8.0
	% censoring	28.2

For reference DNA, buffy coats from 5 anonymous donors were purchased from the Massachusetts General Hospital Blood Bank.

### Validation datasets

Two independent datasets were used for validation. The first dataset included a panel of 160 high-grade serous tumors from UCSF and the Gynecology Oncology Group (UCSF-GOG). These samples were analyzed using a 1 Mb BAC array platform. For these patients, overall survival information was available. The second dataset was obtained from The Cancer Genome Atlas (TCGA) project, included 246 high-grade serous tumors that were analyzed using a custom designed 415 k oligonucleotide array from Agilent. Clinical information for these samples was obtained with permission from the TCGA data committee.

### Oligonucleotide array CGH

High molecular weight genomic DNA was isolated from 72 primary ovarian tumor samples and normal whole blood from 5 anonymous female donors using routine protocol. Array CGH was performed to determine DNA copy number changes using Agilent Human 105 K oligonucleotide microarrays (014698_D_20070820) following the manufacturer's instructions (http://www.home.agilent.com/agilent/home.jspx). Genomic coordinates for this array are based on the NCBI build 36, March 2006 freeze of the assembled human genome (UCSC hg18), available through the UCSC Genome Browser. This array includes a comprehensive probe coverage spanning both coding and non-coding regions, with emphasis on well-known genes, promoters, micro RNAs, and telomeric regions and provides an average spatial resolution of 21.7 kb. Array hybridization, washing and image processing were performed following the protocol described in Gabeau-Lacet et al 2009 [50].

### aCGH data analysis methods

All 5 normal reference DNA samples were hybridized one at a time to identify the common polymorphisms (CNVs) [51]. These CNVs were flagged during image analysis and were eliminated from subsequent analysis. DNA copy number alteration (CNA) was identified through dynamic thresholding of segmented aCGH data. Circular binary segmentation (CBS) was used to segment each hybridization into regions of common mean [52]. For each hybridization, the median absolute deviation (MAD) across all segments was then obtained. Probes assigned to segments with mean value greater than a scaled MAD were identified as gain. Likewise, probes corresponding to segments with mean value less than a scaled MAD were identified as loss. A default MAD scaling factor of 1.11 was utilized for both gains and losses [53]. Both UCSF-GOG and TCGA data sets were subjected to CBS-MAD algorithms followed by GISTIC analysis to identify amplifications and deletions. Following segmentation and classification, data were further reduced, without compromising the continuity and breakpoints, to facilitate downstream analyses [54]. This reduced dataset was used for all subsequent analyses.

To identify minimal regions of common alteration across all hybridizations, the Genomic Identification of Significant Targets in Cancer (GISTIC) approach [55] was utilized on each data set. Threshold selection for the GISTIC procedure was based, conservatively, on the maximum threshold for alteration (across all hybridizations) identified under the MAD approach described above; 0.4 was selected as the gain and loss threshold and 0.25 was selected as the significance threshold. Each analyzed CBS segment consisted of at least four markers. Segments that contained fewer than four markers were combined with the adjacent segment closest in segment value. A q-value was then obtained for each region. Each peak (i.e., region associated with a low q-value) was tested to determine whether the signal was primarily due to broad events, focal events or overlapping events of both types.

Identification of markers associated with survival (PFS and OS) was conducted through utilization of cluster analysis. Unsupervised clustering was first conducted on the set of log2 ratios from the reduced data set described above. Markers on the X chromosome were excluded from the analysis. The Euclidean distance metric was employed in conjunction with the Ward approach for agglomerative clustering. Resultant clusters were then assessed for differences in survival under the Cox proportional hazards model. Because significant differences were identified, GISTIC was performed to identify makers uniquely associated with each subgroup.

To validate the identified set of discriminating markers, supervised clustering was then conducted separately on the UCSF-GOG and TCGA data sets through use of Support Vector Machines [56]; genomic regions in each of the two validation data sets corresponding to the identified discriminating markers were utilized to guide the clustering. For each data set, resultant clusters were then assessed for differences with regard to both overall and progression-free survival.

## Results

### Clinical Characteristics of OVCA patients

The median age at the time of diagnosis of the 72 patient cohort was 60 years (range 37–90) (Table 1). Mean follow up time was 37 months (range 1–212). The majority (93%) of the population presented with advanced stage disease. Surgical staging was utilized as upfront therapy for all patients in the cohort, and this intervention was described as optimal with less than 1 cm of residual disease in 67 patients (88%). Extensive surgical cyto-reduction including peritoneal stripping and bowel resection were utilized in 64% of the cohort in order to achieve an optimal debulking. Only 1 patient did not receive a taxane and platinum-containing regimen as adjuvant therapy after surgery. Six patients were lost to follow up less than 2 months after surgical exploration. Platinum sensitivity defined as a progression free survival of greater than 6 months following the last dose of adjuvant chemotherapy was observed in 42 of 70 (60%) patients, with 12 patients (17%) demonstrating progressive disease despite chemotherapy. Median progression free survival was 8 months, with a median overall survival of 38 months. Univariate survival analysis identified platinum sensitive disease (p<0.0001), optimal cytoreduction (p<0.0001), lack of recurrence or progression (p<0.001) and presenting CA-125<500 U/mL (p<0.04) as prognostic clinical factors predicting an overall survival advantage. A Cox proportional hazards model incorporating these clinical factors adjusted for age revealed that platinum sensitive disease (hazard ratio 0.06), and optimal cytoreduction (0.12) were independent prognostic factors associated with an improved survival.

### Global DNA copy number alterations

**Figure 1:**
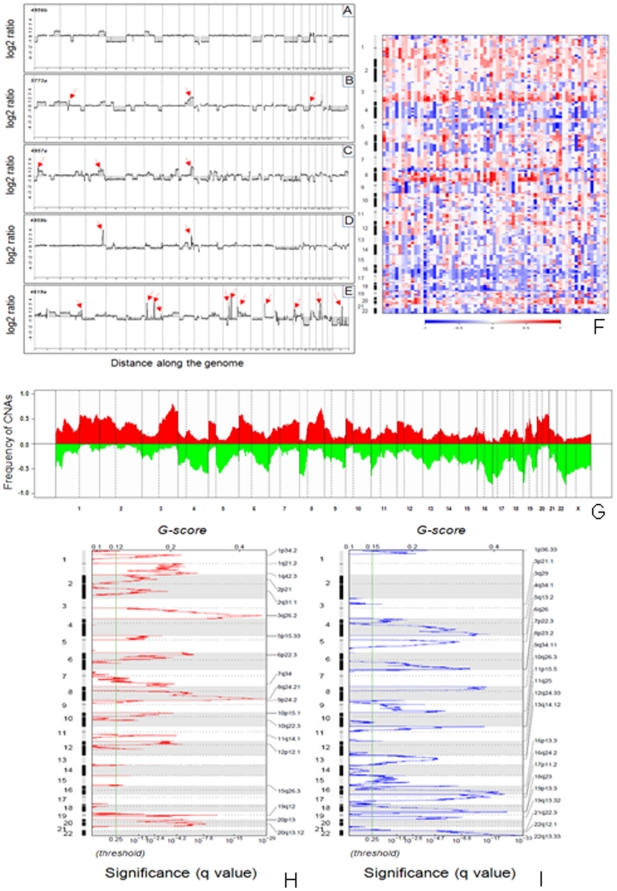
A–E. Representative aCGH profiles of 5 ovarian carcinomas. Log2 ratios (y axis) are plotted along the chromosomes (x axis). Each tumor showing many CNAs including gain and loss of entire chromosome and/or chromosome arms, interstitial deletions, and high-level amplifications (indicated in red arrows). Some tumors had more than 10 high-level amplifications. **F.** Genomic profiles of 72 primary ovarian carcinomas generated by oligonucleotide array CGH. Each column in the left panel represents a tumor sample and rows represent losses and gains of DNA sequences along the length of chromosomes 1 through X as determined by the segmentation analysis of normalized log2 ratios. The color scale ranges from blue (loss) through white (two copies) to red (gain). The right panel indicates the frequencies of gain and loss of oligonucleotide probes on a probe-by-probe basis for all autosomes and the X chromosome. The color scale ranges from white (no changes) to blue (frequent changes). Amplification of 3q26.2 and 8q24.12 including the *EVI1* and *MYC* oncogenes and deletion of 16q24.2 and 22q13.33 were the most frequent alterations observed in 75% and 78% of the ovarian carcinomas respectively. **G.** Overall frequency of CNAs in 72 high-grade serous ovarian carcinomas. **H and I.** GISTIC analysis of copy number gains (**H**) and losses (**I**) in ovarian carcinomas. The statistical significance of the aberrations identified by GISTIC are displayed as false discovery rate q values to account for multiple hypothesis testing (q values; green line is 0.25 cut-off for significance). Scores for each alteration are plotted along the x-axis and the genomic positions are plotted along the y-axis; dotted lines indicate the centromeres. **H**) GISTIC revealed twenty broad and focal regions of gain (copy number threshold = log2 ratio ≥0.4). **I**) Loss of both broad and focal regions were identified by GISTIC (copy number threshold = log2 ratio≤0.4 for broad and ≤0.1 for focal events). Twenty broad and focal regions of losses, including seven focal events, were identified in the background of broad regions. Candidate genes for some broad and focal events are noted. Green stars indicate known or presumed copy number polymorphisms.

Genomic copy number for each probe was determined by calculating the log2 ratio of median signal intensities of the tumor and normal reference DNA. High signal to noise ratios were observed in all samples due to good quality tumor DNA. Representative profiles for five different tumors are shown in Figure 1. A large number of tumors showed some degree of genetic heterogeneity in the background along with distinct increase and decrease of DNA copy numbers involving large portions of chromosome arms (Figure 1A, C, and D). High-level amplifications of regions including 3q26.2 and 8q24.2 were frequently observed (Figure 1B–D). Some tumors displayed more than 10 regions of high-level amplifications (Figure 1E). A genome-wide view of the CNAs in the 72 tumors is shown in Figure 1F and the frequency of amplification and deletion is shown in Figure 1G. In order to identify frequent regions of copy-number alterations, and to define the minimal regions of gains and losses, the statistical method Genomic Identification of Significant Targets In Cancer (GISTIC) was applied to the entire dataset (Figure 1H and I).

GISTIC analysis identified 19 regions of gains along 18 chromosome arms (Figure 1H) and 18 regions of losses along 17 chromosome arms (Figure 1I) distributed throughout the genome. Several chromosomal arms had more than one minimal region of gain and loss. For each alteration, the peak region (i.e., the highest frequency and amplitude of events) was selected as the region most likely to contain a cancer gene. Several oncogenes and tumor suppressor genes previously known to have copy number changes in human ovarian cancer, such as *MYCL1*, *EVI1*, *BRAF*, *MYC*, *KRAS*, *CCNE1*, *TP73*, *RB1*, and *MN1*, were readily identified by GISTIC. Chromosomal locations, frequencies, genomic intervals, gene contents and candidate cancer genes of these changes are highlighted in Table 2. There were 19 regions each of gains and 18 regions of losses (with significant q values) identified with the number of genes ranging from 2–61. The size of deletions ranged from 400 kb to 3 Mb and the number of genes mapping to these regions ranged from 6–106 respectively. In addition, gain and loss of entire chromosome arms were frequently observed. Genes with known or possible function in cancer are highlighted in figure 1H and 1I.

**Table 2:** Amplifications and deletion peaks identified by GISTIC.

Descriptor	Amplification or Deletion	Broad or Focal	Peak Limits	q values	Frequency (%)	Number of genes in peak	Known cancer genes
1p34.2	Amp Peak 1	focal	chr1:39685801–40370914(probes 1602∶1635)	1.58E-05	47	10	MYCL1
1q21.2	Amp Peak 2	broad	chr1:148088286–149154002(probes 4428∶4494)	1.08E-05	54	19	
1q42.3	Amp Peak 3	both	chr1:232669917–234247146(probes 7525∶7574)	3.59E-08	55	7	
2p21	Amp Peak 4	broad	chr2:44361420–47866370(probes 9494∶9634)	0.0008159	47	21	
2q31.1	Amp Peak 5	focal	chr2:175187074–177201863(probes 13436∶13501)	0.0002898	41	16	
3q26.2	Amp Peak 6	both	chr3:170088444–170608075(probes 21389∶21408)	7.20E-28	75	2	EVI1
5p15.33	Amp Peak 7	broad	chr5:763495–848743(probes 28334∶28422)	0.0011277	39	23	TERT
6p22.3	Amp Peak 8	broad	chr6:18594470–21251395(probes 34246∶34314)	1.13E-07	55	21	DEK
7q34	Amp Peak 9	broad	chr7:138546566–139329889(probes 43855∶43891)	9.41E-06	48	61	HIPK2
8q24.21	Amp Peak 10	both	chr8:128870582–129868380(probes 48692∶48710)	4.80E-31	72	6	MYC
9p24.2	Amp Peak 11	broad	chr9:2454035–3357700(probes 49382∶49413)	0.0059345	28	7	
10p15.1	Amp Peak 12	broad	chr10:5337351–6259241(probes 53434∶53478)	1.40E-05	51	18	
10q22.3	Amp Peak 13	focal	chr10:80077917–80824746(probes 55808∶55827)	0.025847	30	19	
11q14.1	Amp Peak 14	focal	chr11:76347688–79590923(probes 60715∶60818)	8.53E-05	34	27	PAK1
12p12.1	Amp Peak 15	broad	chr12:24100724–24946002(probes 63600∶63632)	8.34E-06	42	25	SOX5
19p13.11	Amp Peak 17	broad	chr19:16413980–16621934(probes 86569∶86580)	0.0043441	39	27	NOTCH3
19q12	Amp Peak 18	focal	chr19:34887276–35388638(probes 86927∶86937)	3.99E-11	45	3	CCNE1
20p13	Amp Peak 19	broad	chr20:2081797–3588124(probes 88375∶88451)	1.06E-06	50	26	
20q13.12	Amp Peak 20	focal	chr20:43063207–44606609(probes 89762∶89861)	1.34E-10	58	2	ZMYND8
1p36.33	Del Peak 1	focal	chr1:823965–2511264(probes 10∶101)	0.0002071	46	56	TP73
4q34.1	Del Peak 2	both	chr4:174091952–174549004(probes 27547∶27566)	1.28E-11	55	10	
5q13.2	Del Peak 3	broad	chr5:72832600–75235131(probes 30065∶30149)	2.93E-07	53	29	
6q26	Del Peak 4	broad	chr6:162719313–165363813(probes 38878:38948)	1.84E-08	52	28	
7p22.3	Del Peak 5	focal	chr7:902447–1887560(probes 39172:39224)	0.0046966	39	23	MAD1L1
8p23.2	Del Peak 6	broad	chr8:1422246–3652163(probes 44761:44852)	1.14E-11	60	6	
9q34.11	Del Peak 7	broad	chr9:130311520–131652310(probes 52720:52791)	6.85E-06	46	31	
11p15.5	Del Peak 8	both	chr11:1–562228(probes 57848:57879)	2.58E-11	56	22	HRAS
13q14.12	Del Peak 9	broad	chr13:39671016–49044112(probes 68180:68577)	3.87E-05	49	53	RB1
15q13.1	Del Peak 10	broad	chr15:26364997–27222402(probes 74201:74215)	0.0024529	30	10	
16p13.3	Del Peak 11	focal	chr16:479088–756440(probes 77335:77357)	2.93E-07	51	22	
16q24.2	Del Peak 12	broad	chr16:86172468–87009930(probes 80015:80041)	3.65E-15	78	12	FBXO31, BANP
17p11.2	Del Peak 13	broad	chr17:17622694–18869071(probes 80901:80951)	1.14E-12	65	35	
18q23	Del Peak 14	broad	chr18:71478691–74906480(probes 85605:85715)	1.12E-08	55	12	
19p13.3	Del Peak 15	both	chr19:353214–3505632(probes 85779:85940)	3.99E-16	70	106	
19q13.32	Del Peak 16	broad	chr19:52180116–52242321(probes 87658:87660)	8.45E-08	50	57	
22q12.1	Del Peak 17	broad	chr22:26250112–26828858(probes 92440:92459)	1.86E-09	55	17	MN1
22q13.33	Del Peak 18	both	chr22:48814623–49204003(probes 93575:93600)	3.40E-21	78	30	

Amplification of 3q26.2 including *EVI1* gene and 8q24.12 including *MYC* oncogene were the most frequent alterations occurring in 72–75% of tumors suggesting a role for these genes in tumor maintenance or dissemination process. The most frequently deleted regions (78%) were located on 16q24.2 including *FBXO31* and *BANP* genes and on 22q13.33 (Table 2). Other amplified regions were observed in 28–58% of tumors and deleted regions were observed in 30–70% of tumors respectively. In addition to the identification of regions of gain and loss common to the entire set of tumors, it was also of interest to identify regions of copy number alteration significantly associated with differences in OS and PFS which was assessed using clustering algorithms.

### Cluster analysis

**Figure 2:**
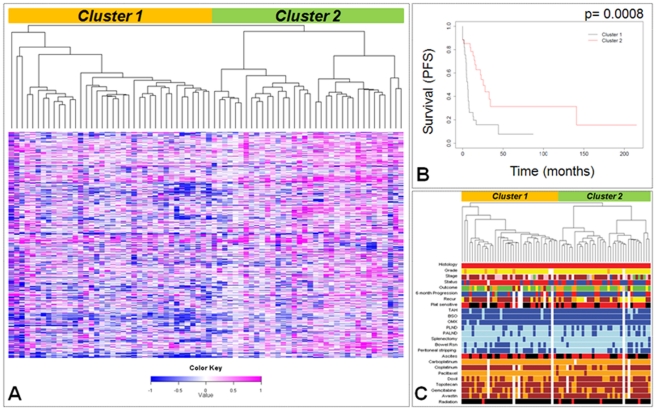
Unsupervised hierarchical clustering of CNAs identifies distinct patient subgroups. **A**) Unsupervised hierarchical clustering of raw log2 ratios derived from 72 serous type ovarian cancers. Copy number values are color coded as follows: blue (loss), white (normal) and magenta (gain). The pattern of dendrogram suggests two major genomic subgroups within the grade 3 tumors. **B**) PFS Kaplan-Meier plot for the two subgroups. **C**) Comparison of clinical characteristics between the patient subgroups. Histology: red = serous; Grade: orange = grade 2, yellow = grade 3; Stage: red = Ic, blue = II, green = IIc, yellow = IIIa, orange = IIIb, brown = IIIc, pink = IV, dark gray = IVa; Status: red = evidence of disease, blue = no evidence of disease; Outcome: green = complete remission, orange = progression, yellow = partial remission, brown = lost to follow up, pink = benign; 6 month progression: red = yes, blue = no, green = P (progression); Recurrence: brown = yes, orange = persistent disease, yellow = no; Platinum response: red = sensitive, black = resistant; Drug: blue = yes, light blue = no; Ascites: red = yes, black = no; Chemo: orange = yes, brown = no; Radiation: red = yes, black = no; General: white = n/a and/or blank.

In order to identify a robust genomic signature and to define clinically relevant genetic subgroups among the high-grade tumors, we applied unsupervised hierarchical clustering algorithm to unfiltered aCGH data from 72 serous type tumors. Figure 2A illustrates the two subgroups that resulted from unsupervised clustering. The two primary subgroups were shown to differ significantly with regard to progression free survival (PFS) (p = 0.0008) and a marginal difference in OS (p = 0.07); figure 2B shows the PFS Kaplan-Meier plot for the two groups. Figure 2C illustrates differences between clusters with regard to clinical covariates. Formal comparison under the Cox proportional hazards model revealed a significant difference between the two subgroups with regards to platinum sensitivity (p = 0.016) and peritoneal stripping (p = 0.011).

**Figure 3:**
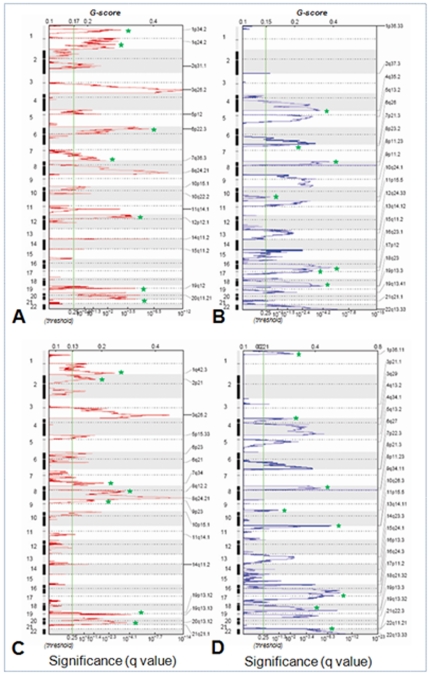
GISTIC analysis of patient subgroups. **A–B**) Cluster 1; **C–D**) Cluster 2 amplification and deletion peaks defined by GISTIC in two patient subgroups show clear difference in the location of peaks. Green stars indicate major differences between the two subgroups. Probes from these regions were used to build the model for training.

To identify CNAs associated with each subgroup, and to determine whether these markers predict outcome independent of grade, we conducted a separate GISTIC analysis of grade 3 tumors only from each cluster. Figure 3A and B show amplifications and deletions identified by GISTIC for tumors in cluster 1 (worse prognosis) and 3C and D for tumors in cluster 2 (better prognosis) respectively. Amplification and deletion peaks unique to each group were readily identified by GISTIC and are indicated by green stars. We used these unique probe sets, listed in Supplementary Table S1, to build a prediction model for conducting supervised clustering. We then evaluated the model against our tumor panel, including grades 2 and 3, using leave-one-out cross validation method. This resulted in 80% accuracy rate in classifying the tumors into good and poor outcome subgroups.

### Validation of independent datasets

**Figure 4:**
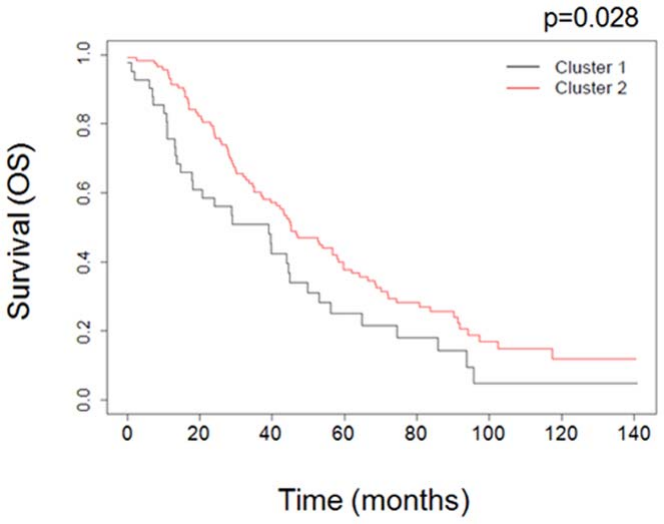
Validation of classification accuracy in UCSF-GOG dataset. Kaplan-Meier plot for UCSF-GOG subgroups identified through supervised clustering. Subgroups are clinically distinct with regard to overall survival (p = 0.028).

Two independent datasets of high-grade serous tumors with clinical follow up information were used for validation. The UCSF-GOG dataset included 160 high-grade tumors, with overall survival information, randomly selected from the Gynecology Oncology Group. Copy number information for this dataset was generated using a 1 Mb BAC array. Data were analyzed using CBS-MAD followed by GISTIC (Figure S3). In order to perform a proper comparison, we pulled targets from the BAC array corresponding to unique probe sets identified from our analysis as described in methods (Table S2 1ists BACs used for clustering). Supervised clustering using our discriminating markers resulted in two subgroups with a statistically significant difference in overall survival (p = 0.028) (Figure 4). Since validation datasets were generated using different array formats, frequency of amplifications and deletions were compared in all three datasets prior to analysis (Table S3).

**Figure 5:**
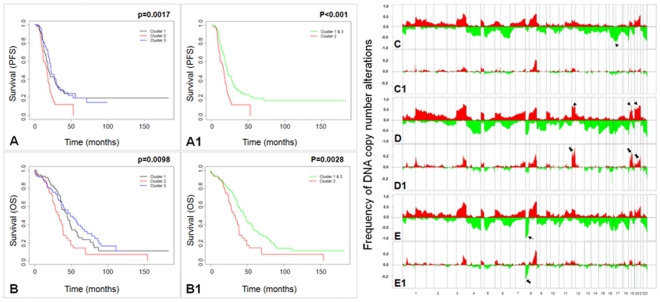
Validation of classification accuracy in TCGA dataset. Kaplan-Meier plots for TCGA subgroups identified through supervised clustering. Subgroups are clinically distinct with regard to both (**A**) progression-free survival (p = 0.0017) and (**B**) overall survival (p = 0.0098). The combined cluster of subgroup 1 and 3 is clinically distinct from subgroup 2 with regard to both (**A1**) progression-free survival (p<0.001) and (**B1**) overall survival (p = 0.0028). **C–E**) Frequencies of genome copy number gain and loss plotted as a function of genome location from 1pter to 22qter in the three clusters identified in the TCGA dataset. Vertical lines indicate chromosome boundaries, and vertical dashed lines indicate position of centromeres along the chromosomes. Positive and negative values indicate frequencies of tumors showing copy number increases (gain shown in red) and decreases (loss shown in green). **C1–E1**) Frequencies of tumors showing high-level amplifications and homozygous deletions in the three TCGA clusters. Data are displayed as described in C–E. Arrows indicate genomic regions where the three clusters differ significantly.

The second dataset included 246 high-grade serous tumors from the TCGA project that were analyzed by a custom made Agilent 415 K oligonucleotide array. Supervised clustering using our discriminating markers resulted in three subgroups with significant difference in PFS (p = 0.0017) and in OS (p = 0.0098) (Figure 5A and B) (Figure S1). Further analysis of the subgroups showed a difference in PFS (p<0.001) and OS (p = 0.0028) between subgroup 2 and combined subgroups 1 and 3 (Figure 5A1 and B1) suggesting that cluster 2 includes patients with worst outcome. Results from the GISTIC analysis of TCGA clusters are shown in Figure S2 A–F. Note that the amplification and deletion peaks of original cluster 1 resembled the amplification and deletion peaks of TCGA cluster 2. To identify genetic alterations specific to each group, we compared CNAs in each cluster (Figure 5C–E1). The TCGA clusters were distinctly different at 8 genomic regions along 8p21.3, 8p23.2, 12p12.1, 17p11.2, 17p12, 19q12, 20q11.21, and 20q13.2.

## Discussion

In this study, we first evaluated global DNA copy number alterations in a panel of 72 clinically annotated high-grade serous ovarian carcinomas to identify specific genetic alterations associated with clinical outcome. Unsupervised hierarchical clustering identified two distinct genomic subgroups with significant difference in clinical outcome. Unique genomic regions identified from each group were then able to successfully divide two independent datasets into clinically distinct subgroups with a significant difference in survival.

Previous studies that attempted to identify the molecular determinants of clinical outcome have focused on single genes because of the frequent involvement of these genes/pathways in serous type ovarian cancers [57], [58]. However, these genes, although frequently associated in ovarian carcinomas, failed to predict outcome compared to the conventional clinical indicator such as the extent of surgery [59], [60]. Gene expression based studies have been useful in predicting clinical phenotypes such as histologic types and stage for various tumor types [61], including breast [62], [63] and ovarian cancers [64], [65], [66], [67], [68].

Several groups have applied aCGH-based genomic technology to identify CNA patterns predictive of platinum resistance [23], [45], and to identify potential driver genes contributing towards ovarian cancer pathogenesis [29], [30], [39]. However, these studies have not established a correlation between CNA pattern and clinical endpoints such as PFS and OS. Some limitations that could have affected the outcome of these studies are sample size, a heterogeneous mixture of samples from different histology/grades, difficulty in combining data from various platforms due to minimal overlap of the results, and lack of a robust dataset for validation. To our knowledge, our study is the first to link a distinct set of CNAs to clinically relevant patient subgroups of high-grade serous ovarian cancers with a significant difference in PFS and OS.

Based on GISTIC analysis, we identified a set of discriminating markers from a cohort a 72 high-grade serous ovarian cancer. Next, we applied those discriminating markers on a dataset generated from a cohort of 160 high-grade serous cancers that were analyzed using a 1 Mb BAC array and identified three clusters which is likely due to larger sample size. Analysis of the three resulting clusters showed a significant difference in overall survival between cluster 1 and combined clusters 2 and 3 (p = 0.028) (Figure 4) (Figures S4 and S5). We then used a cohort of 246 tumors from TCGA that were analyzed using Agilent 415 k oligonucleotide arrays. Using the same discriminating markers, we identified three clusters with a significant difference in both PFS (p = 0.0017) and OS (p = 0.0098) (Figure 5). To further define the groups, we compared the groups in combination. Combination of clusters 1 and 2 versus cluster 3 showed a marginally significant p value of 0.048 for PFS and 0.077 for OS. However, comparison of cluster 2 versus clusters 1 and 3 resulted in a significant difference both in PFS (p<0.001) and OS (p = 0.0028) (Figure 5). Of note, alterations in the cluster 1 of our dataset resembled the alterations in the cluster 1 of UCSF-GOG dataset and cluster 2 of TCGA dataset further confirming our initial results.

In order to identify markers specific for each group, we utilized TCGA dataset since it provided the highest resolution and larger sample size. First, we compared the frequency of losses, gains and high-level amplifications and deletions in each cluster (Figure 5 C–E1). The three TCGA clusters were distinctly different at 8 genomic regions along 8p21.3, 8p23.2, 12p12.1, 17p11.2, 17p12, 19q12, 20q11.21, and 20q13.2. In Cluster 1, 70–76% of samples showed loss of 17p11.2 (Chr17:17646236–21720090) and 17p12 (Chr17:10689461–16833125). In cluster 2, 65–70% of samples had amplifications on 12p12.1 (Chr12:16803022–25998952), 19q12 (Chr19:34794890–35592893), 20q11.21 (Chr20:29363673–29773184) and 20q13.12 (Chr20:42510865–45356897). In cluster 3, 84–94% of tumors showed losses on 8p21.3 (Chr8:22388473–25606748) and 8p23.2 (Chr8:1422246–5781946) regions respectively. Furthermore, in all three datasets the poor outcome subgroups had amplifications along 12p12.1, 19q12 and 20q. In the UCSF-GOG dataset, the 12p12.1 in cluster 1 was distinctly visible compared to the 19q and 20q amplifications. This is likely due to the lower resolution of the array used for these samples. Similarly, the deletions along 8p and 17p were also present in high frequencies in the other two clusters (Supplementary Figure S4).

The minimal region of deletions including homozygous deletions along 17p included the mitogen-activated protain kinase 3 (*MAP2K3*) and mitogen-activated protein kinase 4 (*MAP2K4*) genes. MAP2K3 is activated by mitogenic and environmental stress, and participates in the MAP kinase-mediated signaling cascade. MAP2K4 is a central mediator in the stress activated protein kinase signaling pathway that responds to a number of cellular and environmental stress factors [69]. By phosphorylating MAP kinases such as JNK, MAP2K4 can ultimately transmit stress signals to nuclear transcription factors that mediate various processes including proliferation, apoptosis and differentiation. The majority of metastatic ovarian cancers show significantly reduced expression suggesting that MAP2K4 protein levels are down regulated when cells acquire the ability to grow at a metastatic site [70]. Analysis of a number human ovarian cancer cell lines showed that MAP2K4 expression is not detectable in 3 cell lines (SHOV3ip.1, SKOV-3 and HEY-A8) known to be metastatic in vivo while other members of the MAP2K4 pathway are intact including MEKK1, MKK7, JNK and c-JUN. In addition, key members of the p38 pathway including MKK6, MKK3 and p38 were also present. These results implicate dysregulation of the stress-activated protein kinase signaling cascade in ovarian cancer metastasis and support the hypothesis that MAP2K4 regulates metastatic colonization in ovarian cancer. Several studies have reported somatic mutations in the *MAP2K4* gene in multiple cancer types including ovarian cancer [71], [72], [73]. Kan et al. 2010 stably expressed MAP2K4 mutants in mammalian cells to test their transforming activity. They found that several of the mutants promoted anchorage- independent growth. However, a majority of the MAP2K4 mutants showed reduced activity compared with wild-type kinase. These results suggest that the MAP2K4 mutants may function in a dominant-negative manner and promote anchorage-independent growth in a manner similar to a synthetic dominant-negative MAP2K4 previously reported [74]. From a translational perspective, this finding suggests that modulation of the MAP2K4 pathway, either by restoration of MAP2K4 function alone or in combination with therapeutic agents, could have a clinical benefit.

The second cluster included the worse outcome subgroup. In this cluster, four regions along 12p12.1, 19q12, 20q11.21, and 20q13.12 were amplified in significantly high proportion of samples (Figure 5). The peak region on 12p12.1 included 4 genes: SRY (sex determining region Y)-box 5 isoform b (*SOX5*), (branched chain aminotransferase 1, cytosolic) *BCAT1*, cancer susceptibility candidate 1 isoform a (*CASC1*), and c-K-ras2 protein isoform a precursor (*KRAS*). The *SOX5* gene encodes a member of the SOX (SRY-related HMG-box) family of transcription factors involved in the regulation of embryonic development and in the determination of the cell fate. The encoded protein may act as a transcriptional regulator after forming a protein complex with other proteins [75]. The functional consequence of SOX5 amplification in human cancers has not been explored. One report suggests that over expression of SOX5 enhances nasopharyngeal carcinoma progression and correlates with poor survival [76]. However, its role in ovarian cancer is unexplored.

The Bcat1 gene was isolated in mouse by a subtraction/coexpression strategy with Myc-induced tumors of transgenic mice, and was shown that Bcat1 is a direct genetic target for Myc regulation in mouse [77]. The Bcat1 gene is highly expressed early in embryogenesis, and during organogenesis its expression is localized to the neural tube, the somites, and the mesonephric tubules. The gene is also expressed in several MYC-based tumors. As in mouse, the BCAT1 gene is a target for MYC activity in the oncogenesis process in human [77]. Using expression profiling, Ju et al. 2009 reported differential expression of BCAT1 gene in chemoresistant ovarian cancer compared to chemosensitive tumors [78]. Depletion of BCAT1 by RNA interference in nasopharyngeal cancer cells effectively blocked the proliferation of cells suggesting a role for BCAT1 in tumorigenesis [79]. In colorectal cancer immuno-histochemical analysis of BCAT1 protein showed significantly higher levels of expression in tumor tissues with distant metastasis compared to those without and was shown to be highly predictive of distant metastasis [80]. The Casc1 gene was identified as a strong candidate lung tumor susceptibility gene through whole genome analyses in inbred mice [81].

About 20–40% of human tumors carry mutation in *KRAS* [82]. The *Kras* ^G12D^ conditional knock-in mouse model has been extensively used to study the mechanisms of Ras-induced tumor development [83], [84]. The conditional expression *Kras* ^G12D^ in mice, when combined with other mutations, leads to malignant tumorigenesis in various tissues, including ovarian surface epithelium (OSE). The responses of cells to RAS activation appear to be context dependent such that cells may either undergo oncogenic transformation or become senescent [85]. Although there are rare documented cases of RAS mutations in serous carcinomas, the amplification of this gene may ultimately activate the same pathways that mutant RAS turns on. A better understanding of the molecular targets of RAS in OSE will help identify potential therapeutic targets.

The region on 19q12 included focal amplification of the cyclin E1 (*CCNE1*) gene. High-levels of CCNE1 protein, an activating subunit of the cyclin dependent kinase 2 (CDK2), are often observed in patients with ovarian cancer [86]. Deregulation of cell cycle control is thought to be a prerequisite for tumor development, and several studies have shown an accelerated entry into S phase because of constitutive expression of CCNE1 [87], [88]. Furthermore, CCNE1 is able to induce chromosome instability by inappropriate initiation of DNA replication, and centrosome duplication [89]. Amplification of *CCNE1* in ovarian cancer correlates with drug resistance [23] and poor clinical outcome [90]. Our finding confirmed the above-mentioned studies and identified amplification of *CCNE1* as a marker of poor outcome and a possible therapeutic target.

Amplification of two distinct regions on 20q11.21, and 20q13.12 were associated with the poor outcome subgroup. The region on 20q11.21 included two notable genes among others: inhibitor of DNA binding 1 (*ID1*) and BCL2-like 1 (*BCL2L1*). ID1 is a member of a family of 4 proteins (ID1-4) known to inhibit the activity of basic helix loop helix transcription factors by blocking their ability to bind DNA. ID1 has been implicated in a variety of cellular processes including cell growth, differentiation, angiogenesis, and neoplastic transformation. It has been shown that ID1 is de-regulated in multiple cancers and up-regulation of ID1 is correlated with high-grades and poor prognosis in human cancers [91], [92]. ID1 has also been shown to be an effector of the p53-dependent DNA damage response pathway [93]. In ovarian cancer, the level of Id1 protein expression correlates with malignant potential, associated with poor differentiation and aggressive behavior of tumor leading to poor clinical outcome [94]. *BCL2L1* is a BCL2-related gene and can function as a BCL2-independent regulator of programmed cell death [95]. Both *BCL2* and *BCL2L1* are antiapoptotic and downstream targets of p53. Overexpression of *BCL2L1* suppresses mitochondrial-mediated apoptosis and enhances cancer cell survival in cancer models [96]. Several studies report the expression of *BCL2L1* in 60–70% of ovarian cancer and that *BCL2L1* expression is associated with chemoresistant and recurrent disease [97].

Previous studies using conventional CGH have reported consistent high-level amplification of the 20q13.12 region encompassing many genes that may play causal role in ovarian cancer pathogenesis [42], [98], [99]. In this study, we have identified a 2.8 Mb region including 61 genes. Among others, the likely candidates are *MMP9*, *PI3*, *NCOA5*, *TP53RK*, *ZMYD8* [100], [101], [102], [103], [104]. Based on integrated analysis of DNA copy number and expression profiling results, 20q11.22–q13.12 region has been reported to be associated with poor response to primary treatment [23]. More recently, another study using tissue microarray composed of late stage, high-grade serous ovarian carcinomas correlated *PI3* expression with poor overall survival [101].

Finally, cluster 3 samples predominantly showed losses on 8p21.3 and 8p23.2 regions. Several candidate tumor suppressor genes that are less known to be implicated in human cancers include *DOCK5* [105] and *CSMD1* [106] map to this region. Based on the available literature, the above mentioned genes are likely to play important roles but future studies are required to define their roles in the pathogenesis of serous type ovarian carcinomas.

Whether expressions of all candidate genes described above are altered in high grade serous ovarian cancer is not yet known and is currently under investigation in our laboratory. Our study may also have missed rare copy number variants, including duplications and deletions, in predisposing cancer susceptibility genes since the normal reference DNA was made from healthy donors but not matched normal DNA from each patient. However, it is less likely given the very large deletions and amplifications we identified in these tumors.

In summary, the results from this study illustrate the unique molecular landscape of the genetic subgroups that exist within the high-grade tumors. In the future, using these genomic markers, the high-grade serous tumors can be stratified into clinically relevant subgroups, help develop new diagnostic strategies and eventually lead to targeted therapy.

## Supporting Information

Figure S1 Supervised clustering of TCGA samples.
(TIF)

Figure S2 GISTIC analysis of TCGA clusters.
(TIF)

Figure S3 DNA copy number analysis of UCSF-GOG samples. **A.** Summary of CNAs in UCSF-GOG dataset. **B–C.** GISTIC analysis of UCSF-GOG dataset.
(TIF)

Figure S4 Supervised clustering of UCSF-GOG dataset.
(TIF)

Figure S5 Kaplan-Meier analysis of UCSF-GOG clusters.
(TIF)

Table S1 Probe set corresponding to amplification and deletion peaks used for supervised clustering.
(DOCX)

Table S2 List of BACs used for clustering GOG data.
(DOC)

Table S3 Amplifications and deletion peaks identified by GISTIC.
(DOCX)

## References

[1] Ozols RF, Bookman MA, Connolly DC, Daly MB, Godwin AK (2004). Focus on epithelial ovarian cancer.. Cancer Cell.

[2] Cannistra SA (2004). Cancer of the ovary.. N Engl J Med.

[3] Jemal A, Siegel R, Xu J, Ward E (2010). Cancer statistics, 2010.. CA Cancer J Clin.

[4] Dinh P, Harnett P, Piccart-Gebhart MJ, Awada A (2008). New therapies for ovarian cancer: cytotoxics and molecularly targeted agents.. Crit Rev Oncol Hematol.

[5] Balvert-Locht HR, Coebergh JW, Hop WC, Brolmann HA, Crommelin M (1991). Improved prognosis of ovarian cancer in The Netherlands during the period 1975–1985: a registry-based study.. Gynecol Oncol.

[6] Agarwal R, Kaye SB (2003). Ovarian cancer: strategies for overcoming resistance to chemotherapy.. Nat Rev Cancer.

[7] FIGO Cancer Committee (1986). Staging announcement.. Gynecol Oncol.

[8] Mutch DG (2002). Surgical management of ovarian cancer.. Semin Oncol.

[9] Ozols RF, Bundy BN, Greer BE, Fowler JM, Clarke-Pearson D (2003). Phase III trial of carboplatin and paclitaxel compared with cisplatin and paclitaxel in patients with optimally resected stage III ovarian cancer: a Gynecologic Oncology Group study.. J Clin Oncol.

[10] du Bois A, Neijt JP, Thigpen JT (1999). First line chemotherapy with carboplatin plus paclitaxel in advanced ovarian cancer–a new standard of care?. Ann Oncol.

[11] Biagi JJ, Eisenhauer EA (2003). Systemic treatment policies in ovarian cancer: the next 10 years.. Int J Gynecol Cancer.

[12] Neijt JP, Engelholm SA, Tuxen MK, Sorensen PG, Hansen M (2000). Exploratory phase III study of paclitaxel and cisplatin versus paclitaxel and carboplatin in advanced ovarian cancer.. J Clin Oncol.

[13] Sandercock J, Parmar MK, Torri V, Qian W (2002). First-line treatment for advanced ovarian cancer: paclitaxel, platinum and the evidence.. Br J Cancer.

[14] Perez RP (1998). Cellular and molecular determinants of cisplatin resistance.. Eur J Cancer.

[15] Niedner H, Christen R, Lin X, Kondo A, Howell SB (2001). Identification of genes that mediate sensitivity to cisplatin.. Mol Pharmacol.

[16] Li M, Balch C, Montgomery JS, Jeong M, Chung JH (2009). Integrated analysis of DNA methylation and gene expression reveals specific signaling pathways associated with platinum resistance in ovarian cancer.. BMC Med Genomics.

[17] Seidman JD, Horkayne-Szakaly I, Haiba M, Boice CR, Kurman RJ (2004). The histologic type and stage distribution of ovarian carcinomas of surface epithelial origin.. Int J Gynecol Pathol.

[18] Landen CN, Birrer MJ, Sood AK (2008). Early events in the pathogenesis of epithelial ovarian cancer.. J Clin Oncol.

[19] Karst AM, Drapkin R (2010). Ovarian cancer pathogenesis: a model in evolution.. J Oncol.

[20] Bonome T, Lee JY, Park DC, Radonovich M, Pise-Masison C (2005). Expression profiling of serous low malignant potential, low-grade, and high-grade tumors of the ovary.. Cancer Res.

[21] Zorn KK, Bonome T, Gangi L, Chandramouli GV, Awtrey CS (2005). Gene expression profiles of serous, endometrioid, and clear cell subtypes of ovarian and endometrial cancer.. Clin Cancer Res.

[22] Jazaeri AA, Awtrey CS, Chandramouli GV, Chuang YE, Khan J (2005). Gene expression profiles associated with response to chemotherapy in epithelial ovarian cancers.. Clin Cancer Res.

[23] Etemadmoghadam D, deFazio A, Beroukhim R, Mermel C, George J (2009). Integrated genome-wide DNA copy number and expression analysis identifies distinct mechanisms of primary chemoresistance in ovarian carcinomas.. Clin Cancer Res.

[24] Haverty PM, Hon LS, Kaminker JS, Chant J, Zhang Z (2009). High-resolution analysis of copy number alterations and associated expression changes in ovarian tumors.. BMC Med Genomics.

[25] Slamon DJ, Godolphin W, Jones LA, Holt JA, Wong SG (1989). Studies of the HER-2/neu proto-oncogene in human breast and ovarian cancer.. Science.

[26] Baker VV, Borst MP, Dixon D, Hatch KD, Shingleton HM (1990). c-myc amplification in ovarian cancer.. Gynecol Oncol.

[27] Shayesteh L, Lu Y, Kuo WL, Baldocchi R, Godfrey T (1999). PIK3CA is implicated as an oncogene in ovarian cancer.. Nat Genet.

[28] Nanjundan M, Nakayama Y, Cheng KW, Lahad J, Liu J (2007). Amplification of MDS1/EVI1 and EVI1, located in the 3q26.2 amplicon, is associated with favorable patient prognosis in ovarian cancer.. Cancer Res.

[29] Cheng KW, Lahad JP, Kuo WL, Lapuk A, Yamada K (2004). The RAB25 small GTPase determines aggressiveness of ovarian and breast cancers.. Nat Med.

[30] Park JT, Li M, Nakayama K, Mao TL, Davidson B (2006). Notch3 gene amplification in ovarian cancer.. Cancer Res.

[31] Katoh M (2008). Cancer genomics and genetics of FGFR2 (Review).. Int J Oncol.

[32] Brown LA, Irving J, Parker R, Kim H, Press JZ (2006). Amplification of EMSY, a novel oncogene on 11q13, in high grade ovarian surface epithelial carcinomas.. Gynecol Oncol.

[33] Kohler MF, Marks JR, Wiseman RW, Jacobs IJ, Davidoff AM (1993). Spectrum of mutation and frequency of allelic deletion of the p53 gene in ovarian cancer.. J Natl Cancer Inst.

[34] Singer G, Oldt R, Cohen Y, Wang BG, Sidransky D (2003). Mutations in BRAF and KRAS characterize the development of low-grade ovarian serous carcinoma.. J Natl Cancer Inst.

[35] Santin AD, Zhan F, Bellone S, Palmieri M, Cane S (2004). Gene expression profiles in primary ovarian serous papillary tumors and normal ovarian epithelium: identification of candidate molecular markers for ovarian cancer diagnosis and therapy.. Int J Cancer.

[36] Yu Y, Xu F, Peng H, Fang X, Zhao S (1999). NOEY2 (ARHI), an imprinted putative tumor suppressor gene in ovarian and breast carcinomas.. Proc Natl Acad Sci U S A.

[37] Bruening W, Prowse AH, Schultz DC, Holgado-Madruga M, Wong A (1999). Expression of OVCA1, a candidate tumor suppressor, is reduced in tumors and inhibits growth of ovarian cancer cells.. Cancer Res.

[38] Mok SC, Chan WY, Wong KK, Muto MG, Berkowitz RS (1996). SPARC, an extracellular matrix protein with tumor-suppressing activity in human ovarian epithelial cells.. Oncogene.

[39] Gorringe KL, Ramakrishna M, Williams LH, Sridhar A, Boyle SE (2009). Are there any more ovarian tumor suppressor genes? A new perspective using ultra high-resolution copy number and loss of heterozygosity analysis.. Genes Chromosomes Cancer.

[40] Pinkel D, Segraves R, Sudar D, Clark S, Poole I (1998). High resolution analysis of DNA copy number variation using comparative genomic hybridization to microarrays.. Nat Genet.

[41] Arnold N, Hagele L, Walz L, Schempp W, Pfisterer J (1996). Overrepresentation of 3q and 8q material and loss of 18q material are recurrent findings in advanced human ovarian cancer.. Genes Chromosomes Cancer.

[42] Sonoda G, Palazzo J, du Manoir S, Godwin AK, Feder M (1997). Comparative genomic hybridization detects frequent overrepresentation of chromosomal material from 3q26, 8q24, and 20q13 in human ovarian carcinomas.. Genes Chromosomes Cancer.

[43] Gray JW, Suzuki S, Kuo WL, Polikoff D, Deavers M (2003). Specific keynote: genome copy number abnormalities in ovarian cancer.. Gynecol Oncol.

[44] Caserta D, Benkhalifa M, Baldi M, Fiorentino F, Qumsiyeh M (2008). Genome profiling of ovarian adenocarcinomas using pangenomic BACs microarray comparative genomic hybridization.. Mol Cytogenet.

[45] Kim SW, Kim JW, Kim YT, Kim JH, Kim S (2007). Analysis of chromosomal changes in serous ovarian carcinoma using high-resolution array comparative genomic hybridization: Potential predictive markers of chemoresistant disease.. Genes Chromosomes Cancer.

[46] Nowee ME, Snijders AM, Rockx DA, de Wit RM, Kosma VM (2007). DNA profiling of primary serous ovarian and fallopian tube carcinomas with array comparative genomic hybridization and multiplex ligation-dependent probe amplification.. J Pathol.

[47] Gorringe KL, Jacobs S, Thompson ER, Sridhar A, Qiu W (2007). High-resolution single nucleotide polymorphism array analysis of epithelial ovarian cancer reveals numerous microdeletions and amplifications.. Clin Cancer Res.

[48] Sotiriou C, Piccart MJ (2007). Taking gene-expression profiling to the clinic: when will molecular signatures become relevant to patient care?. Nat Rev Cancer.

[49] Straehle C, Cardoso F, Azambuja E, Dolci S, Meirsman L (2009). Better translation from bench to bedside: breakthroughs in the individualized treatment of cancer.. Crit Care Med.

[50] Gabeau-Lacet D, Engler D, Gupta S, Scangas GA, Betensky RA (2009). Genomic profiling of atypical meningiomas associates gain of 1q with poor clinical outcome.. J Neuropathol Exp Neurol.

[51] Zhang L, Huang J, Yang N, Liang S, Barchetti A (2006). Integrative genomic analysis of protein kinase C (PKC) family identifies PKCiota as a biomarker and potential oncogene in ovarian carcinoma.. Cancer Res.

[52] Olshen AB, Venkatraman ES, Lucito R, Wigler M (2004). Circular binary segmentation for the analysis of array-based DNA copy number data.. Biostatistics.

[53] Korkola JE, Heck S, Olshen AB, Reuter VE, Bosl GJ (2008). In vivo differentiation and genomic evolution in adult male germ cell tumors.. Genes Chromosomes Cancer.

[54] van de Wiel MA, van Wieringen WN (2007). CGHregions: dimension reduction for array CGH data with minimal informatics loss.. Cancer Informatics.

[55] Beroukhim R, Getz G, Nghiemphu L, Barretina J, Hsueh T (2007). Assessing the significance of chromosomal aberrations in cancer: methodology and application to glioma.. Proc Natl Acad Sci U S A.

[56] Dimitriadou E, Hornik K, Leisch F, Meyer D, Weingessel A (2006).

[57] Merritt WM, Lin YG, Han LY, Kamat AA, Spannuth WA (2008). Dicer, Drosha, and outcomes in patients with ovarian cancer.. N Engl J Med.

[58] Farley J, Fuchiuji S, Darcy KM, Tian C, Hoskins WJ (2009). Associations between ERBB2 amplification and progression-free survival and overall survival in advanced stage, suboptimally-resected epithelial ovarian cancers: a Gynecologic Oncology Group Study.. Gynecol Oncol.

[59] Griffiths CT (1975). Surgical resection of tumor bulk in the primary treatment of ovarian carcinoma.. Natl Cancer Inst Monogr.

[60] Krag KJ, Canellos GP, Griffiths CT, Knapp RC, Parker LM (1989). Predictive factors for long-term survival in patients with advanced ovarian cancer.. Gynecol Oncol.

[61] Ramaswamy S, Golub TR (2002). DNA microarrays in clinical oncology.. J Clin Oncol.

[62] Sorlie T, Tibshirani R, Parker J, Hastie T, Marron JS (2003). Repeated observation of breast tumor subtypes in independent gene expression data sets.. Proc Natl Acad Sci U S A.

[63] Chin K, DeVries S, Fridlyand J, Spellman PT, Roydasgupta R (2006). Genomic and transcriptional aberrations linked to breast cancer pathophysiologies.. Cancer Cell.

[64] Spentzos D, Levine DA, Ramoni MF, Joseph M, Gu X (2004). Gene expression signature with independent prognostic significance in epithelial ovarian cancer.. J Clin Oncol.

[65] Bonome T, Levine DA, Shih J, Randonovich M, Pise-Masison CA (2008). A gene signature predicting for survival in suboptimally debulked patients with ovarian cancer.. Cancer Res.

[66] Berchuck A, Iversen ES, Luo J, Clarke JP, Horne H (2009). Microarray analysis of early stage serous ovarian cancers shows profiles predictive of favorable outcome.. Clin Cancer Res.

[67] Yoshihara K, Tajima A, Komata D, Yamamoto T, Kodama S (2009). Gene expression profiling of advanced-stage serous ovarian cancers distinguishes novel subclasses and implicates ZEB2 in tumor progression and prognosis.. Cancer Sci.

[68] Bell D, Berchuck A, Birrer M, Chien J, Cramer DW (2011). Integrated genomic analyses of ovarian carcinoma.. Nature.

[69] Cuenda A (2000). Mitogen-activated protein kinase kinase 4 (MKK4).. Int J Biochem Cell Biol.

[70] Yamada SD, Hickson JA, Hrobowski Y, Vander Griend DJ, Benson D (2002). Mitogen-activated protein kinase kinase 4 (MKK4) acts as a metastasis suppressor gene in human ovarian carcinoma.. Cancer Res.

[71] Parsons DW, Wang TL, Samuels Y, Bardelli A, Cummins JM (2005). Colorectal cancer: mutations in a signalling pathway.. Nature.

[72] Greenman C, Stephens P, Smith R, Dalgliesh GL, Hunter C (2007). Patterns of somatic mutation in human cancer genomes.. Nature.

[73] Kan Z, Jaiswal BS, Stinson J, Janakiraman V, Bhatt D (2010). Diverse somatic mutation patterns and pathway alterations in human cancers.. Nature.

[74] Cazillis M, Bringuier AF, Delautier D, Buisine M, Bernuau D (2004). Disruption of MKK4 signaling reveals its tumor-suppressor role in embryonic stem cells.. Oncogene.

[75] Lefebvre V, Li P, de Crombrugghe B (1998). A new long form of Sox5 (L-Sox5), Sox6 and Sox9 are coexpressed in chondrogenesis and cooperatively activate the type II collagen gene.. Embo J.

[76] Huang DY, Lin YT, Jan PS, Hwang YC, Liang ST (2008). Transcription factor SOX-5 enhances nasopharyngeal carcinoma progression by down-regulating SPARC gene expression.. J Pathol.

[77] Schuldiner O, Eden A, Ben-Yosef T, Yanuka O, Simchen G (1996). ECA39, a conserved gene regulated by c-Myc in mice, is involved in G1/S cell cycle regulation in yeast.. Proc Natl Acad Sci U S A.

[78] Ju W, Yoo BC, Kim IJ, Kim JW, Kim SC (2009). Identification of genes with differential expression in chemoresistant epithelial ovarian cancer using high-density oligonucleotide microarrays.. Oncol Res.

[79] Zhou W, Feng X, Li H, Wang L, Li H (2007). Functional evidence for a nasopharyngeal carcinoma-related gene BCAT1 located at 12p12.. Oncol Res.

[80] Yoshikawa R, Yanagi H, Shen CS, Fujiwara Y, Noda M (2006). ECA39 is a novel distant metastasis-related biomarker in colorectal cancer.. World J Gastroenterol.

[81] Liu P, Wang Y, Vikis H, Maciag A, Wang D (2006). Candidate lung tumor susceptibility genes identified through whole-genome association analyses in inbred mice.. Nat Genet.

[82] Bos JL (1988). The ras gene family and human carcinogenesis.. Mutat Res.

[83] Johnson L, Mercer K, Greenbaum D, Bronson RT, Crowley D (2001). Somatic activation of the K-ras oncogene causes early onset lung cancer in mice.. Nature.

[84] Dinulescu DM, Ince TA, Quade BJ, Shafer SA, Crowley D (2005). Role of K-ras and Pten in the development of mouse models of endometriosis and endometrioid ovarian cancer.. Nat Med.

[85] Sarkisian CJ, Keister BA, Stairs DB, Boxer RB, Moody SE (2007). Dose-dependent oncogene-induced senescence in vivo and its evasion during mammary tumorigenesis.. Nat Cell Biol.

[86] Farley J, Smith LM, Darcy KM, Sobel E, O'Connor D (2003). Cyclin E expression is a significant predictor of survival in advanced, suboptimally debulked ovarian epithelial cancers: a Gynecologic Oncology Group study.. Cancer Res.

[87] Ohtsubo M, Theodoras AM, Schumacher J, Roberts JM, Pagano M (1995). Human cyclin E, a nuclear protein essential for the G1-to-S phase transition.. Mol Cell Biol.

[88] Resnitzky D, Hengst L, Reed SI (1995). Cyclin A-associated kinase activity is rate limiting for entrance into S phase and is negatively regulated in G1 by p27Kip1.. Mol Cell Biol.

[89] Kawamura K, Izumi H, Ma Z, Ikeda R, Moriyama M (2004). Induction of centrosome amplification and chromosome instability in human bladder cancer cells by p53 mutation and cyclin E overexpression.. Cancer Res.

[90] Nakayama N, Nakayama K, Shamima Y, Ishikawa M, Katagiri A (2010). Gene amplification CCNE1 is related to poor survival and potential therapeutic target in ovarian cancer.. Cancer.

[91] Lin CQ, Singh J, Murata K, Itahana Y, Parrinello S (2000). A role for Id-1 in the aggressive phenotype and steroid hormone response of human breast cancer cells.. Cancer Res.

[92] Schoppmann SF, Schindl M, Bayer G, Aumayr K, Dienes J (2003). Overexpression of Id-1 is associated with poor clinical outcome in node negative breast cancer.. Int J Cancer.

[93] Qian Y, Chen X (2008). ID1, inhibitor of differentiation/DNA binding, is an effector of the p53-dependent DNA damage response pathway.. J Biol Chem.

[94] Schindl M, Schoppmann SF, Strobel T, Heinzl H, Leisser C (2003). Level of Id-1 protein expression correlates with poor differentiation, enhanced malignant potential, and more aggressive clinical behavior of epithelial ovarian tumors.. Clin Cancer Res.

[95] Boise LH, Gonzalez-Garcia M, Postema CE, Ding L, Lindsten T (1993). bcl-x, a bcl-2-related gene that functions as a dominant regulator of apoptotic cell death.. Cell.

[96] Chipuk JE, Fisher JC, Dillon CP, Kriwacki RW, Kuwana T (2008). Mechanism of apoptosis induction by inhibition of the anti-apoptotic BCL-2 proteins.. Proc Natl Acad Sci U S A.

[97] Williams J, Lucas PC, Griffith KA, Choi M, Fogoros S (2005). Expression of Bcl-xL in ovarian carcinoma is associated with chemoresistance and recurrent disease.. Gynecol Oncol.

[98] Iwabuchi H, Sakamoto M, Sakunaga H, Ma YY, Carcangiu ML (1995). Genetic analysis of benign, low-grade, and high-grade ovarian tumors.. Cancer Res.

[99] Tanner MM, Grenman S, Koul A, Johannsson O, Meltzer P (2000). Frequent amplification of chromosomal region 20q12–q13 in ovarian cancer.. Clin Cancer Res.

[100] Lin B, White JT, Wu J, Lele S, Old LJ (2009). Deep depletion of abundant serum proteins reveals low-abundant proteins as potential biomarkers for human ovarian cancer.. Proteomics Clin Appl.

[101] Clauss A, Ng V, Liu J, Piao H, Russo M (2010). Overexpression of elafin in ovarian carcinoma is driven by genomic gains and activation of the nuclear factor kappaB pathway and is associated with poor overall survival.. Neoplasia.

[102] Jiang C, Ito M, Piening V, Bruck K, Roeder RG (2004). TIP30 interacts with an estrogen receptor alpha-interacting coactivator CIA and regulates c-myc transcription.. J Biol Chem.

[103] Peterson D, Lee J, Lei XC, Forrest WF, Davis DP (2010). A chemosensitization screen identifies TP53RK, a kinase that restrains apoptosis after mitotic stress.. Cancer Res.

[104] Colland F, Jacq X, Trouplin V, Mougin C, Groizeleau C (2004). Functional proteomics mapping of a human signaling pathway.. Genome Res.

[105] Feller SM, Lewitzky M (2006). Potential disease targets for drugs that disrupt protein– protein interactions of Grb2 and Crk family adaptors.. Curr Pharm Des.

[106] Kuo KT, Guan B, Feng Y, Mao TL, Chen X (2009). Analysis of DNA copy number alterations in ovarian serous tumors identifies new molecular genetic changes in low-grade and high-grade carcinomas.. Cancer Res.

