# Supplementary figures and images for: Genome Wide DNA Copy Number Analysis of Serous Type Ovarian Carcinomas Identifies Genetic Markers Predictive of Clinical Outcome
Source: PLoS One. 2012 Feb 15;7(2):e30996. doi: 10.1371/journal.pone.0030996 (PMC3280266; doi:10.1371/journal.pone.0030996)

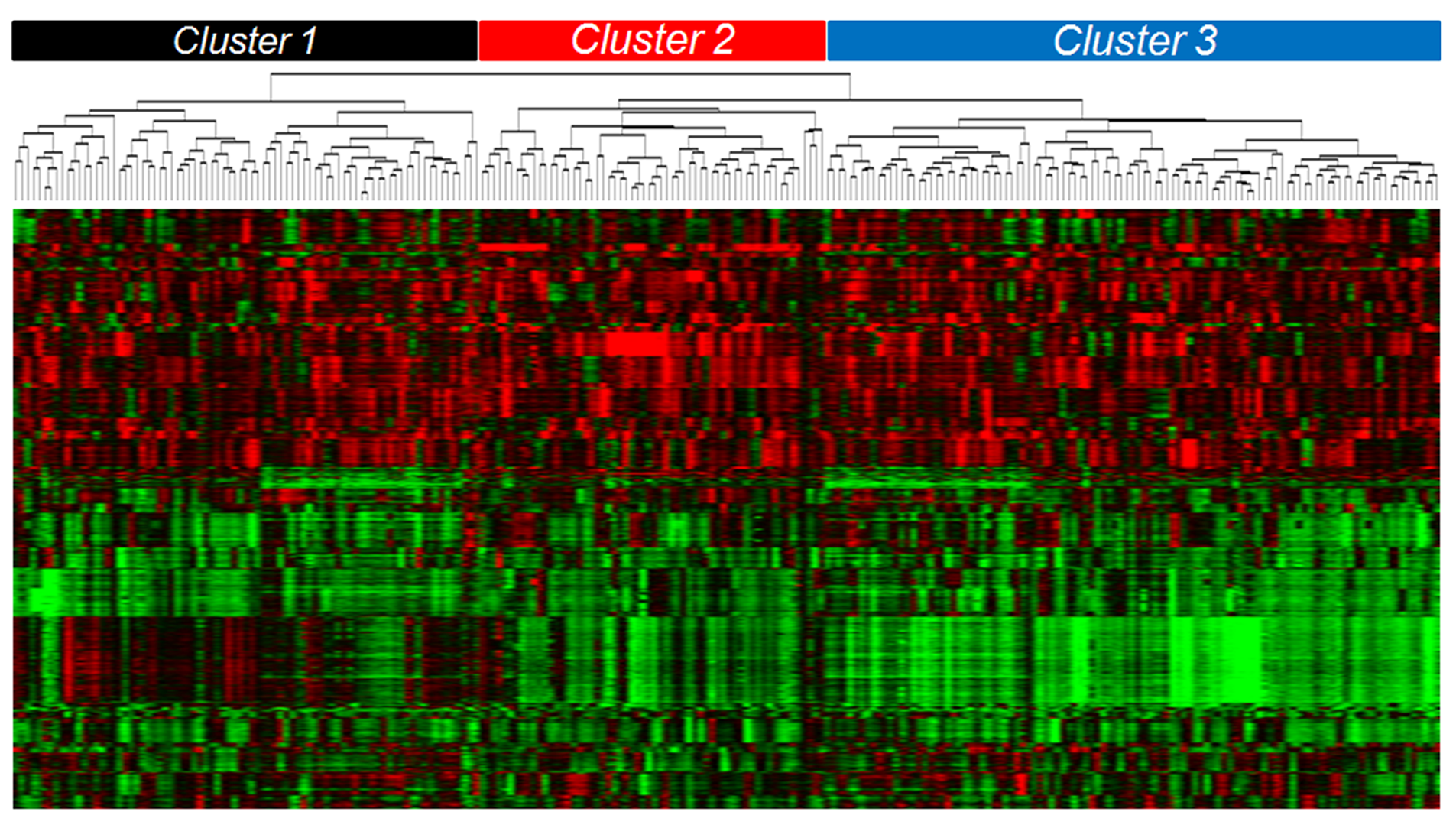

Supplement: Figure S1 — Supervised clustering of TCGA samples. (TIF) [file pone.0030996.s001.tif]

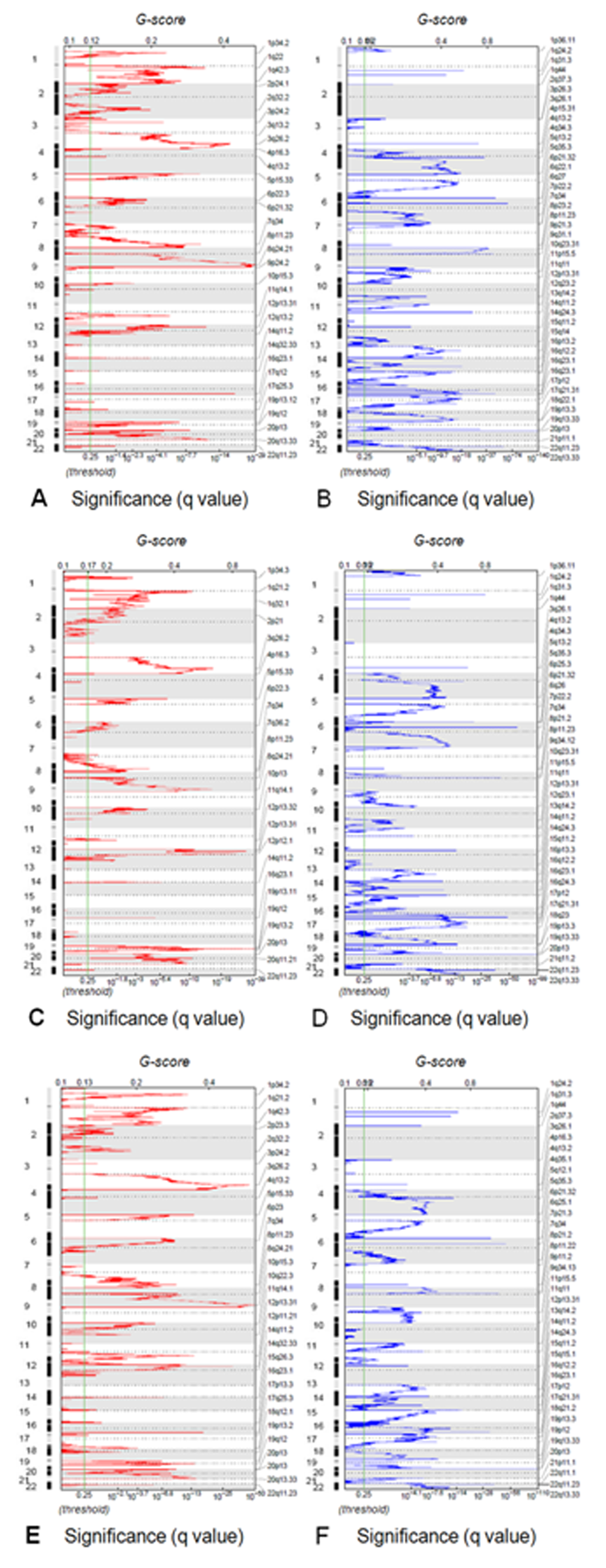

Supplement: Figure S2 — GISTIC analysis of TCGA clusters. (TIF) [file pone.0030996.s002.tif]

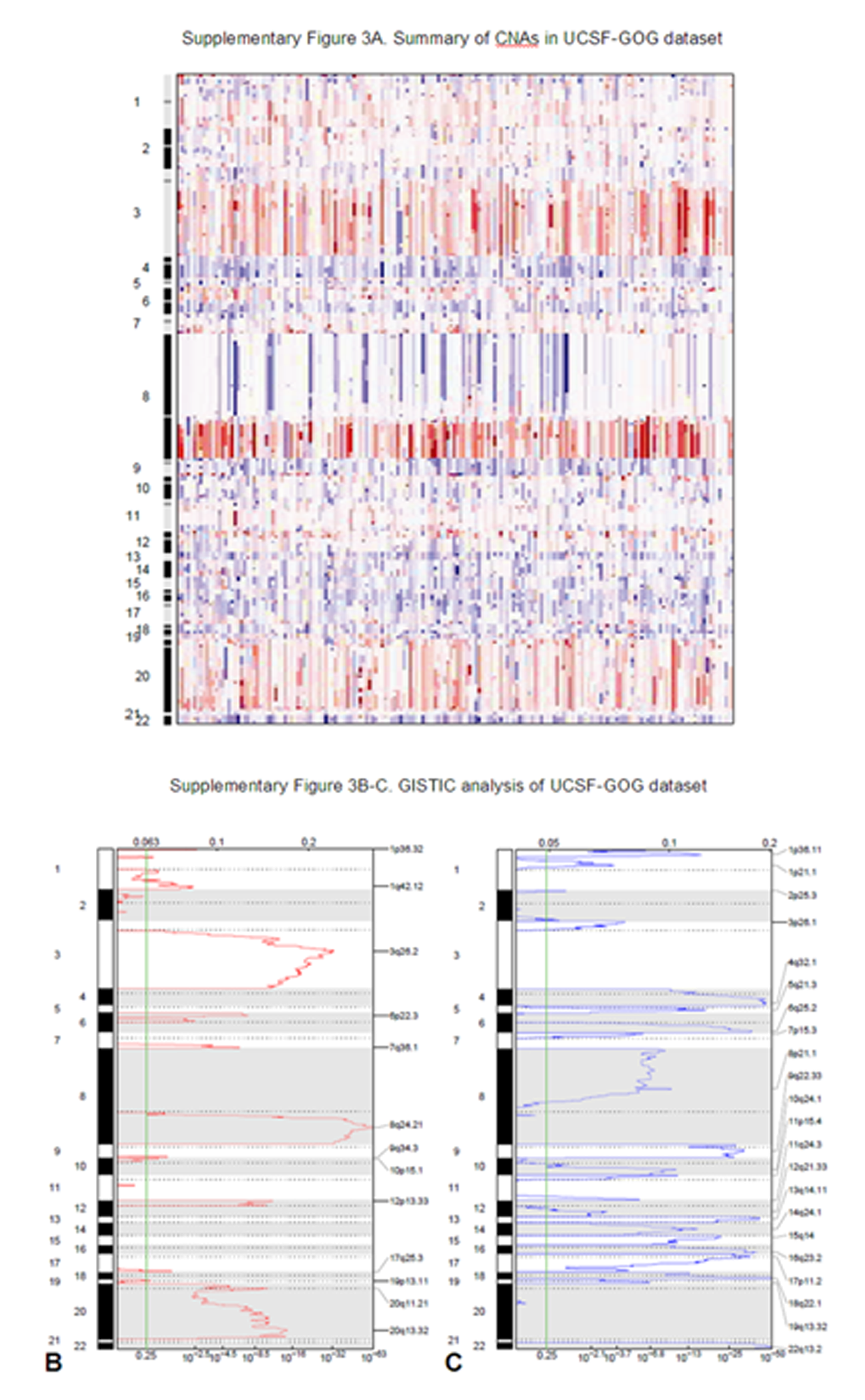

Supplement: Figure S3 — DNA copy number analysis of UCSF-GOG samples. A. Summary of CNAs in UCSF-GOG dataset. B–C. GISTIC analysis of UCSF-GOG dataset. (TIF) [file pone.0030996.s003.tif]

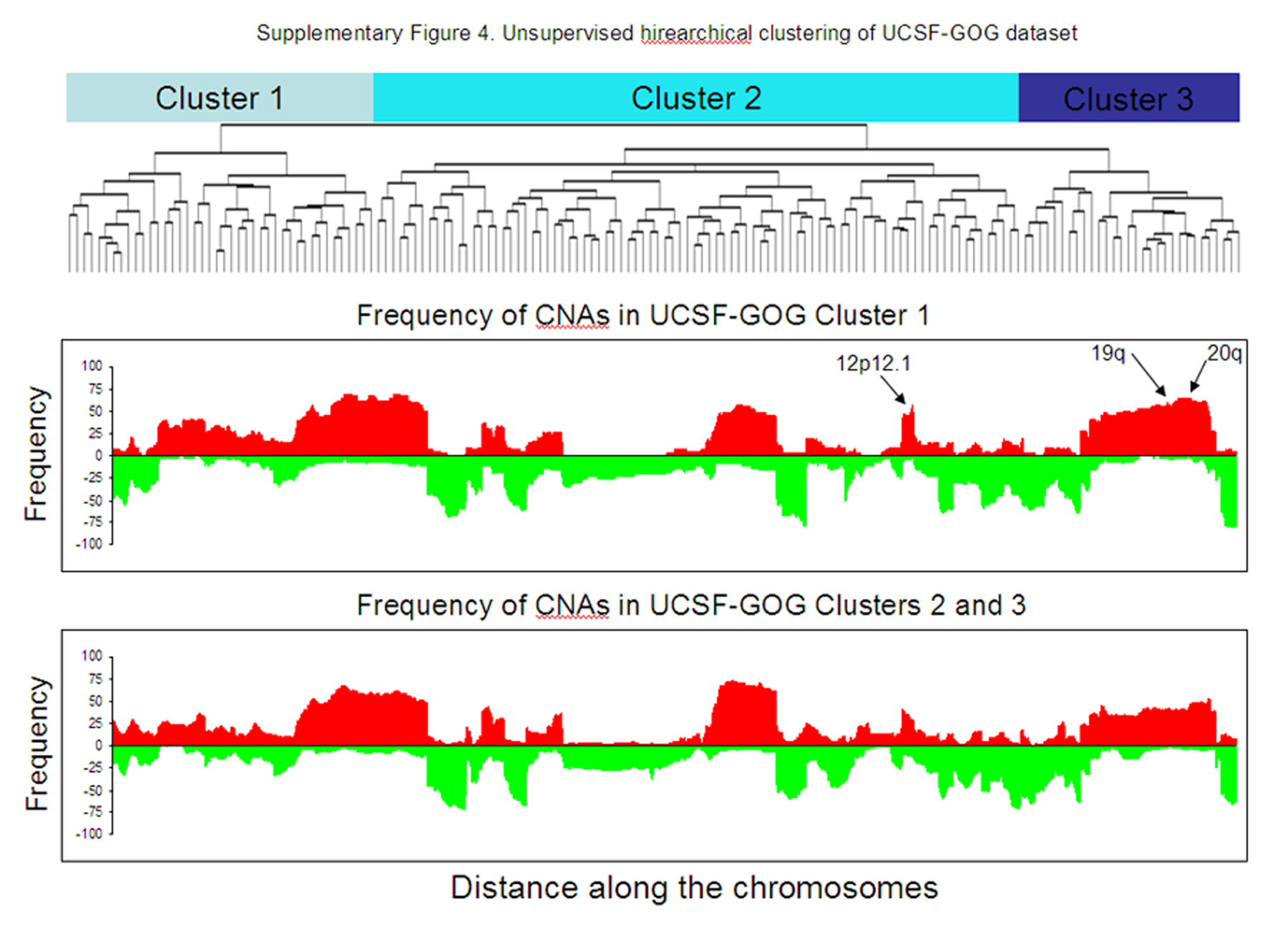

Supplement: Figure S4 — Supervised clustering of UCSF-GOG dataset. (TIF) [file pone.0030996.s004.tif]

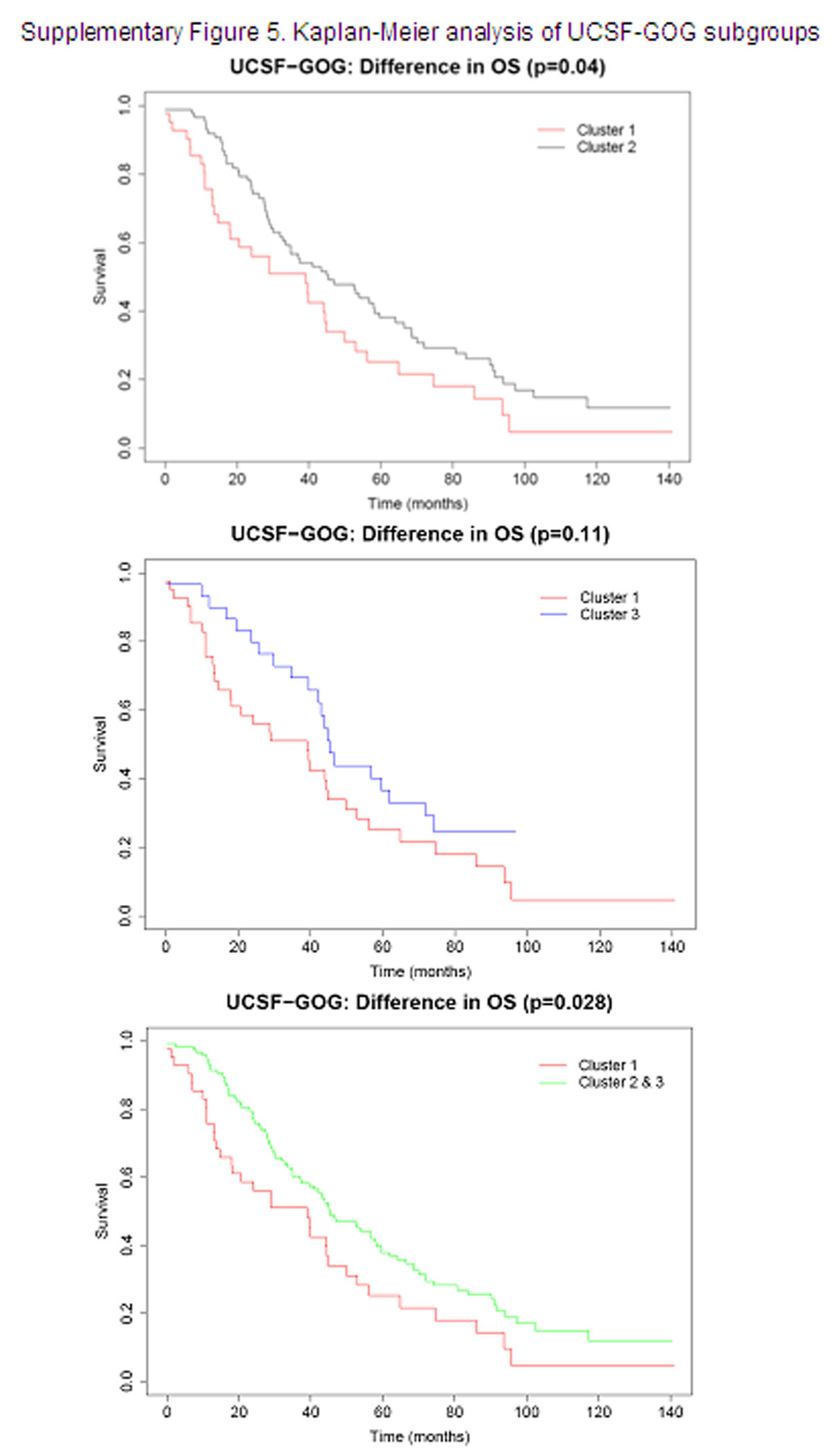

Supplement: Figure S5 — Kaplan-Meier analysis of UCSF-GOG clusters. (TIF) [file pone.0030996.s005.tif]
