# Supplementary material for: Genome Wide DNA Copy Number Analysis of Serous Type Ovarian Carcinomas Identifies Genetic Markers Predictive of Clinical Outcome
Source: PLoS One. 2012 Feb 15;7(2):e30996. doi: 10.1371/journal.pone.0030996 (PMC3280266; doi:10.1371/journal.pone.0030996)
Supplement: Table S1 — Probe set corresponding to amplification and deletion peaks used for supervised clustering. (DOCX) [file pone.0030996.s006.docx]

| Suppl. Table 1. Probe set corresponding to amplification and deletion peaks used for supervised clustering | | | | | |
| --- | --- | --- | --- | --- | --- |
| **Cytogenetic map** | **Start** | **Stop** | **Amplification or Deletion** | **Number of Genes in the Interval** | **Genes of Interest** |
| 1p36.33 | 857406 | 1142357 | Deletion | 10 | HES4 |
| 1p34.2-p34.3 | 39805990 | 40435032 | Amplification | 9 | BMP8A, MYCL1 |
| 1q24.2 | 148123789 | 148545900 | Amplification | 7 |  |
| 1q42.2 | 232809474 | 233848432 | Amplification | 10 | IRF2BP2 |
| 2p21 | 46626081 | 48455702 | Amplification | 11 | TTC7A, EPCAM, FBXO11 |
| 2q31.1 | 176737071 | 177893995 | Amplification | 12 | HOXD cluster |
| 3p21.1 | 51948377 | 51990245 | Deletion | 8 | PARP3 |
| 5p15.33 | 865241 | 1345984 | Amplification | 7 | BRD9, TRIP13, NKD2, hTERT |
| 5q13.2 | 70931240 | 72240180 | Deletion | 8 | MAP1B, TNPO1 |
| 6p22.3 | 20643040 | 21338805 | Amplification | 4 | E2F3, ID4, CDKAL1 |
| 6q26 | 161692918 | 163063425 | Deletion | 3 | PARK2 |
| 6q27 | 168040442 | 169778500 | Deletion | 4 | MLLT4, KIF24, DACT2, SMOC2 |
| 7p22.1 | 4752122 | 5518924 | Deletion | 25 | FOXK1, RADIL, RBAK, WIPI2 |
| 7q33 | 137146419 | 137826289 | Amplification | 18 | CREB3L2 |
| 7q36.3 | 158519999 | 158620979 | Amplification | 16 | SHH |
| 8p23.2 | 2834472 | 4830585 | Deletion | 6 | CSMD1 |
| 8p21.3 | 22388472 | 22539437 | Deletion | 27 | SORBS3, BIN3 |
| 8q12.1 | 61264533 | 62784495 | Amplification | 17 | RAB2, CHD7 |
| 9q34.11 | 130412270 | 131652371 | Deletion | 36 |  |
| 10q26.3 | 133895780 | 135064066 | Deletion | 21 | INPP5A |
| 12p12.1 | 24505529 | 26040166 | Amplification | 28 | SOX5, BCAT1, KRAS |
| 15q24.1 | 71955983 | 72707602 | Deletion | 22 | PML, SIN3A |
| 16q23.1 | 77236437 | 78658712 | Deletion | 2 | WWOX1 |
| 16q24.2 | 86172468 | 87915613 | Deletion | 24 | SNAI3 |
| 17p12 | 11538298 | 11985231 | Deletion | 33 | MAP2K4 |
| 17p11.2 | 19639481 | 21374038 | Deletion | 40 | MAP2K3 |
| 18q21.32 | 55424390 | 55596833 | Deletion | 31 | SOCS6, CCBE1 |
| 18q23 | 74835850 | 74859139 | Deletion | 14 | SALL3 |
| 19p13.12 | 15853126 | 16299359 | Amplification | 11 | RAB8A, NOTCH3 |
| 19q12 | 34794889 | 35220955 | Amplification | 6 | CCNE1 |
| 20q11.21 | 29566769 | 29769473 | Amplification | 10 | BCL2L1, ID1 |
| 20q13.2 | 43061569 | 44606670 | Amplification | 58 | MMP9, PI3, NCOA3 |
| 21q22.3 | 44565059 | 45530518 | Deletion | 25 | ITGB2 |
| 22q11.21 | 18351655 | 19063487 | Deletion | 15 | TBX1 |
