# Supplementary material for: Genome Wide DNA Copy Number Analysis of Serous Type Ovarian Carcinomas Identifies Genetic Markers Predictive of Clinical Outcome
Source: PLoS One. 2012 Feb 15;7(2):e30996. doi: 10.1371/journal.pone.0030996 (PMC3280266; doi:10.1371/journal.pone.0030996)
Supplement: Table S2 — List of BACs used for clustering GOG data. (DOC) [file pone.0030996.s007.doc]

| **Supplementary Table 2. List of BACs used for clustering GOG data** | | |
| --- | --- | --- |
| **Cytoband_Jul03_AL** | **Target** | **Gene/Locus** |
| 1p36.33 | GP-004F09 | LOC51767 |
| 1p36.33 | GP-019H02 | FLJ10346, PRKCZ, SKI |
| 1p36.32 | GP-021F07 | TP73 |
| 1p36.11 | GP-010B06 | NUDC |
| 1p36.11 | GP-020C12 | WASF2 |
| 1p36.11 | GP-010G03 | FGR |
| 1p34.2 | GP-021C11 | SLC2A1 |
| 1p34.2 | GP-009E10 | ESTs |
| 1q22 | GP-027C06 | RAB25 |
| 1q22 | GP-026B12 | RAB25 |
| 1q22 | GP-026A11 | RAB25 |
| 1q22 | GP-026D12 | RAB25 |
| 1q23.1 | GP-027G08 | RAB25 |
| 1q23.1 | GP-007H01 | EST/unknown |
| 1q42.13 | GP-011G07 | ARF1 |
| 1q42.13 | GP-008C04 | KIAA0133 |
| 1q42.2 | GP-014D10 | stSG51125, stSG52299 |
| 1q42.3 | GP-003E06 | TM7SF1 |
| 1q43 | GP-010G08 | CHML |
| 2p22.3 | GP-010A05 | DKFZP564F0522 |
| 2p22.2 | GP-004H10 | FEZ2 |
| 2p21 | GP-004G10 | EPAS1 |
| 2p21 | GP-005B11 | KIAA0671 |
| 2q31.1 | GP-006A11 | SLC25A12 |
| 2q31.1 | GP-007A04 | EST/unknown |
| 2q31.1 | GP-010F03 | EST/X15507 |
| 2q31.1 | GP-010A03 | EST/unknown |
| 3p21 | GP-006A06 | LIMD1 |
| 3p21 | GP-008F07 | PRKAR2A |
| 3p21 | GP-007A06 | RhoA/ARHA |
| 4q34.3 | GP-012D05 | EST/N67140 |
| 4q35.1 | GP-004A10 | FACL2 |
| 4q35.1 | GP-010D11 | EST/AA460002 |
| 4q35.1 | GP-004B09 | SLC25A4 |
| 5p15.33 | GP-019F09 | SEC6, AHRR |
| 5p15.2 | GP-010H01 | EST/AA488655 |
| 5q13.2 | GP-026F08 | CCNB1 |
| 5q13.2 | GP-009D08 | CDK7 |
| 5q13.2 | GP-002D01 | FOXD1 |
| 6p22.3 | GP-020E11 | CAP2 |
| 6p22.3 | GP-001D09 | KLK6 |
| 6p22.3 | GP-020D08 | E2F3 |
| 6q26 | GP-001G09 | LPA |
| 6q26 | GP-006D06 | MAP3K4 |
| 6q27 | GP-007H07 | RPS6KA2 |
| 6q27 | GP-008G08 | EST/unknown |
| 6q27 | GP-020A03 | PDCD2 |
| 7p22.3 | GP-007A10 | MAD1L1 |
| 7p22.1 | GP-008G09 | EST/unknown |
| 7q34 | GP-008F08 | EST/unknown |
| 7q34 | GP-021B12 | BRAF |
| 7q34 | GP-009E09 | KEL |
| 7q34 | GP-005C12 | CASP2 |
| 7q36.1 | GP-021A08 | H91620p/PRKAG2 |
| 7q36.2 | GP-007A08 | EST/unknown |
| 7q36.3 | GP-020A10 | VIPR2 |
| 8p23.3 | GP-023A04 |  |
| 8p23.3 | GP-025B01 |  |
| 8p23.3 | GP-025C03 |  |
| 8p23.3 | GP-024C09 |  |
| 8p23.3 | GP-022F09 |  |
| 8p23.3 | GP-025A04 |  |
| 8p21.3 | GP-023H11 |  |
| 8p21.3 | GP-023H07 |  |
| 8p21.3 | GP-023D11 |  |
| 8p21.3 | GP-009D09 | EST/unknown |
| 8p21.3 | GP-022C04 |  |
| 8q12.2 | GP-010G12 | EST/AA259140 |
| 8q12.2 | GP-001E11 | LPL |
| 9p23 | GP-009D11 | hbrm |
| 9p23 | GP-001E04 | SMARCA2 |
| 9q34.11 | GP-008C01 | EST/unknown |
| 9q34.11 | GP-004D05 | TOR1B |
| 10q21.1 | GP-003A04 | TRABID |
| 10q21.1 | GP-008A03 | EST/unknown |
| 10q21.2 | GP-010D08 | EST/N93171 |
| 10q21.3 | GP-011A05 | EGR2 |
| 10q22.2 | GP-011H06 | KIAA0974 |
| 10q22.3 | GP-013D02 | EST/AA044244 |
| 10q23.32 | GP-008C05 | EST/unknown |
| 10q24.1 | GP-008H06 | EST/unknown |
| 10q24.2 | GP-012A06 | EST/N26801 |
| 10q26.3 | GP-009C10 | EST/unknown |
| 10q26.3 | GP-019C08 | CYP2E |
| 10q26.3 | GP-020A12 | DUX4 |
| 12p12.3 | GP-003A03 | ARHGDIB |
| 12p12.1 | GP-001E05 | CMAS |
| 12p12.1 | GP-006B01 | KRAS2 |
| 12p12.1 | GP-013C06 | DKFZp434O0227. |
| 14q23.2 | GP-013D01 | (KIAA1011) SYNE-2 |
| 14q23.3 | GP-013B09 | EST/W81100 |
| 15q24.1 | GP-004H05 | NEO1 |
| 15q24.1 | GP-008F11 | FLJ20374 |
| 15q24.3 | GP-008F02 | KIAA1055 |
| 16p13.3 | GP-012B11 | EST/AA526259 |
| 16p13.3 | GP-019D08 | HBA1 |
| 16q23.1 | GP-003D04 | MAF |
| 16q23.2 | GP-007C07 | EST/unknown |
| 16q23.3 | GP-007A01 | EST/unknown |
| 16q24.3 | GP-004A11 | FANCA |
| 16q24.3 | GP-019D11 | DPEP1, CPNE7, PCOLN3, CDK10, C16orf7 |
| 17p12 | GP-003C03 | EBBP/TRIM16 |
| 17p11.2 | GP-002H03 | KRT17 |
| 17p11.2 | GP-007G09 | EST/unknown |
| 18q21.32 | GP-009B06 | SCOP (KIAA0606) |
| 18q21.32 | GP-007C05 | FOP |
| 18q21.32 | GP-006E03 | PI8/SERPINB8 |
| 18q23 | GP-002G05 | NFATC1 |
| 18q23 | GP-019F06 | KIAA0863, PARD6G |
| 19p13.12 | GP-006D11 | PIK3R2 |
| 19q13.12 | GP-007F03 | EST/unknown |
| 19q13.31 | GP-007F04 | XRCC1 |
| 19q13.31 | GP-006C12 | KCNN4 |
| 20q11.21 | GP-019A11 |  |
| 20q11.21 | GP-013A01 | BCL2L1 |
| 20q11.21 | GP-018F10 |  |
| 20q11.21 | GP-021C09 | HCK |
| 20q13.12 | GP-018C05 |  |
| 20q13.12 | GP-021E09 | MYBL2 |
| 20q13.12 | GP-018D02 |  |
| 20q13.12 | GP-018H12 |  |
| 20q13.12 | GP-019B11 |  |
| 20q13.12 | GP-021E10 | NCOA3 |
| 20q13.12 | GP-018B01 |  |
| 20q13.12 | GP-019A01 |  |
| 21q21.1 | GP-005G02 | BTG3 |
| 21q22.2 | GP-026G07 | ETS2 |
| 21q22.3 | GP-026E01 | MX2 |
| 22q11.21 | GP-026F01 | [CDC45L](http://www.ncbi.nlm.nih.gov/mapview/maps.cgi?org=hum&chr=22&MAPS=genec,ugHs,genes-r&cmd=focus&fill=40&query=uid(7791)&QSTR=cell cycle*) |
| 22q11.21 | GP-026B06 | CRKL |
