# Supplementary material for: Genome Wide DNA Copy Number Analysis of Serous Type Ovarian Carcinomas Identifies Genetic Markers Predictive of Clinical Outcome
Source: PLoS One. 2012 Feb 15;7(2):e30996. doi: 10.1371/journal.pone.0030996 (PMC3280266; doi:10.1371/journal.pone.0030996)
Supplement: Table S3 — Amplifications and deletion peaks identified by GISTIC. (DOCX) [file pone.0030996.s008.docx]

| **Supplementary Table 3. Amplifications and deletion peaks identified by GISTIC** | | | | | |
| --- | --- | --- | --- | --- | --- |
| **Descriptor** | **Amplification or Deletion** | **Peak Limits** | **MGH Dataset Frequency (%)** | **GOG Dataset Frequency (%)** | **TCGA Dataset Frequency (%)** |
| 1p34.2 | Amp Peak 1 | chr1:39685801-40370914 | 47 | 26 | 31 |
| 1q21.2 | Amp Peak 2 | chr1:148088286-149154002 | 54 | 30 | 47 |
| 1q42.3 | Amp Peak 3 | chr1:232669917-234247146 | 55 | 33 | 36 |
| 2p21 | Amp Peak 4 | chr2:44361420-47866370 | 47 | 21 | 24 |
| 2q31.1 | Amp Peak 5 | chr2:175187074-177201863 | 41 | 23 | 23 |
| 3q26.2 | Amp Peak 6 | chr3:170088444-170608075 | 75 | 66 | 70 |
| 5p15.33 | Amp Peak 7 | chr5:763495-848743 | 39 | 17 | 38 |
| 6p22.3 | Amp Peak 8 | chr6:18594470-21251395 | 55 | 39 | 31 |
| 7q34 | Amp Peak 9 | chr7:138546566-139329889 | 48 | 30 | 34 |
| 8q24.21 | Amp Peak 10 | chr8:128870582-129868380 | 72 | 65 | 69 |
| 9p24.2 | Amp Peak 11 | chr9:2454035-3357700 | 28 | 13 | 15 |
| 10p15.1 | Amp Peak 12 | chr10:5337351-6259241 | 51 | 21 | 30 |
| 10q22.3 | Amp Peak 13 | chr10:80077917-80824746 | 30 | 10 | 16 |
| 11q14.1 | Amp Peak 14 | chr11:76347688-79590923 | 34 | 11 | 30 |
| 12p12.1 | Amp Peak 15 | chr12:24100724-24946002 | 42 | 34 | 41 |
| 19p13.11 | Amp Peak 17 | chr19:16413980-16621934 | 39 | 26 | 32 |
| 19q12 | Amp Peak 18 | chr19:34887276-35388638 | 45 | 23 | 40 |
| 20p13 | Amp Peak 19 | chr20:2081797-3588124 | 50 | 35 | 40 |
| 20q13.12 | Amp Peak 20 | chr20:43063207-44606609 | 58 | 45 | 42 |
| 1p36.33 | Del Peak 1 | chr1:823965-2511264 | 46 | 28 | 20 |
| 4q34.1 | Del Peak 2 | chr4:174091952-174549004 | 55 | 64 | 52 |
| 5q13.2 | Del Peak 3 | chr5:72832600-75235131 | 53 | 37 | 55 |
| 6q26 | Del Peak 4 | chr6:162719313-165363813 | 52 | 64 | 45 |
| 7p22.3 | Del Peak 5 | chr7:902447-1887560 | 39 | 29 | 31 |
| 8p23.2 | Del Peak 6 | chr8:1422246-3652163 | 60 | 25 | 61 |
| 9q34.11 | Del Peak 7 | chr9:130311520-131652310 | 46 | 56 | 34 |
| 11p15.5 | Del Peak 8 | chr11:1-562228 | 56 | 43 | 41 |
| 13q14.12 | Del Peak 9 | chr13:39671016-49044112 | 49 | 56 | 44 |
| 15q13.1 | Del Peak 10 | chr15:26364997-27222402 | 30 | 50 | 37 |
| 16p13.3 | Del Peak 11 | chr16:479088-756440 | 51 | 42 | 29 |
| 16q24.2 | Del Peak 12 | chr16:86172468-87009930 | 78 | 60 | 54 |
| 17p11.2 | Del Peak 13 | chr17:17622694-18869071 | 65 | 61 | 61 |
| 18q23 | Del Peak 14 | chr18:71478691-74906480 | 55 | 61 | 52 |
| 19p13.3 | Del Peak 15 | chr19:353214-3505632 | 70 | 30 | 60 |
| 19q13.32 | Del Peak 16 | chr19:52180116-52242321 | 50 | 34 | 32 |
| 22q12.1 | Del Peak 17 | chr22:26250112-26828858 | 55 | 60 | 49 |
| 22q13.33 | Del Peak 18 | chr22:48814623-49204003 | 78 | 68 | 70 |
